# Potential opportunities and challenges of utilizing brewer’s spent grain in sustainable fish feeding within the circular economy

**DOI:** 10.3389/fphys.2026.1766656

**Published:** 2026-03-18

**Authors:** E. Geremia, E. Buonocore, P. Venditti, I. Adamos, S. Paolacci, C. Agnisola, G. Napolitano

**Affiliations:** 1 “Environment, Resources and Sustainable Development”, Department of Science and Technology, Parthenope University of Naples, Centro Direzionale, Naples, Italy; 2 Department of Biology, Federico II University of Naples, Complesso Universitario Monte Sant’Angelo, Naples, Italy; 3 AquaBioTech Group, Mosta, Malta

**Keywords:** BSG, circular economy, fish nutrition, life cycle assessment, oxidative stress, sustainable aquafeed

## Abstract

Alongside growing concern about the environmental impacts of the aquaculture production sector, increasing interest is being directed toward sustainable alternatives to traditional protein sources used in aquafeed, such as fish meal and soybean meal. Among these, brewers’ spent grain (BSG), a byproduct of the brewing industry, has recently emerged as an alternative ingredient. BSG is a low-cost, year-round resource that may be well-suited for the circular economy. It contains moderate levels of proteins (15%–27% of dry matter) and appreciable amounts of antioxidant compounds (polyphenols and phenolic acids). Several studies show that BSG can replace up to 50% of traditional protein sources in aquafeed formulations without compromising growth performance. However, BSG has drawbacks, including its high content of antinutritional factors (ANFs) and its relatively low level of essential amino acids. Processing strategies such as hydrolysis and fermentation appear promising for reducing ANFs and enhancing protein availability. Moreover, information on the global impacts of using BSG in aquafeed, either raw or processed, remains limited. A Life Cycle Assessment analysis conducted on two hypothetical diets with 10% or 37% BSG as partial replacements for traditional ingredients reveals that a detailed assessment of environmental impacts across all stages of aquafeed production is essential to support the large-scale adoption of BSG in aquaculture.

## 1 Introduction

Brewers’ spent grain (BSG), the primary by-product of beer production, is a widely available waste that can be valorized as a functional ingredient in animal feed thanks to its high fiber and protein content. In recent years, research has increasingly explored the potential applications of BSG in aquaculture, particularly within the frameworks of sustainability and the circular economy. A keyword-based literature analysis of publications on “brewers’ spent grain” since 1953 (see Supplementary Figure S1 in Supplementary Material S1) highlights a growing scientific interest in its innovative use within aquatic food systems. This trend is largely driven by the urgent need to identify more sustainable alternatives to conventional aquafeed ingredients. At present, aquaculture relies heavily on fishmeal and fish oil derived from wild fisheries, resources associated with significant environmental pressures and increasing economic costs.

In this context, BSG represents a promising bioresource capable of transforming brewery waste into a valuable input for aquafeed production, thereby contributing to a more circular and resource-efficient value chain. The partial replacement of fish-derived ingredients with BSG could alleviate pressure on marine ecosystems, reduce feed costs, and improve the overall sustainability profile of farmed fish products (Karlsen and Skov, 2022).

Despite these promising prospects, the incorporation of BSG into aquaculture feeds poses several challenges. Its effective integration within a circular economy framework requires addressing issues such as limited digestibility and ensuring the nutritional adequacy of BSG-based diets for various fish species, with respect to growth performance, product quality, and animal health. Furthermore, it is essential to evaluate whether BSG inclusion genuinely reduces the environmental impact of feed production, taking into account factors such as biomass transportation, processing energy requirements, and associated emissions. Comprehensive life cycle assessments are, therefore, necessary to substantiate the sustainability claims related to BSG utilization.

This review provides a comprehensive synthesis of current knowledge on the use of brewers’ spent grain as an aquafeed ingredient. It examines the expected benefits, including reduced dependence on marine-derived inputs, potential cost savings, and enhanced waste valorization, while also addressing the scientific and practical challenges that must be overcome to enable large-scale adoption. Understanding these factors is crucial for assessing the feasibility of BSG as a sustainable and scalable component of future aquaculture feeding strategies.

## 2 The rise of aquaculture production and related environmental impacts

Following remarkable growth since the 1950s, the fisheries and aquaculture sector reached a record production of 224 million tons in 2022, of which 83% consisted of aquatic animals (FAO, 2024). Currently, the fisheries and aquaculture sector supplies approximately 15% of global animal protein requirements, with contributions exceeding 50% in several regions in Asia and Africa (FAO, 2024). This growth was mainly sustained by aquaculture (MacLeod et al., 2019; FAO, 2024), which now accounts for 65% of the sector’s total revenue. In contrast, fishery production has stagnated due to increasing pressure on fish stocks caused by overfishing, pollution, inadequate management, and other factors (FAO, 2024).

Although aquaculture production is expected to continue growing, concerns about its environmental impacts are also rising due to its waste generation and resource use, especially water and energy (Ahmad et al., 2022; Jiang et al., 2022). A key aspect of aquaculture development is the rising demand for fish feed production, which is essential for ensuring both production efficiency and product quality (Mitra, 2021). Fish feeds must supply adequate levels of unsaturated fatty acids and crude protein (CP), typically ranging from 25% to 55%, depending on species-specific feeding habits (herbivorous, carnivorous, or omnivorous) and life stage (Webster and Lim, 2002; Boyd, 2015). Fishmeal (FM) and fish oil (FO) have traditionally fulfilled these nutritional requirements, due to their high digestibility and nutritional quality (FAO, 2024). They are derived from a variety of wild-caught marine fish species, including Peruvian anchoveta (*Engraulis ringens* Jenyns 1842), herring (*Clupea* spp.), pollock (*Pollachius* spp.), sand eels (*Hyperoplus* spp.), menhaden (*Brevoortia* spp.), and several sardine genera (Directorate General for Maritime Affairs and Fisheries and EUMOFA, 2020; Stickney and Gatlin, 2022; FAO, 2024).

As aquaculture continues to expand and wild fish stocks remain under pressure, reducing the sector’s dependence on marine-derived resources has become imperative (Kok et al., 2020; Karlsen and Skov, 2022). This shift requires the identification and adoption of alternative ingredients to replace FM and FO. Concerns regarding FM and FO extend beyond the depletion of fish stocks, encompassing the significant environmental impacts associated with their entire production chain, from fishing to processing. Different studies using the Life Cycle Assessment (LCA) approach (ISO, 2006), which enable comparison of environmental impacts across farms and feed formulations (Ziegler et al., 2003; Hospido and Tyedmers, 2005; Thrane, 2006; Cao et al., 2013; Prescott, 2017; Ghamkhar and Hicks, 2020), have shown that fish harvesting and transportation account for 60%–90% of the total greenhouse gas (GHG) emissions associated with FM and FO production. Moreover, the processing stage requires substantial amounts of heavy fuel oil or natural gas to power the fish cooking, drying and general plant operations. Additional environmental impacts arise from processing residues of FM and FO, including suspended solids and oil fractions, which are often discharged into the marine ecosystem (Fréon et al., 2017). On the other hand, the use of fishing by-products as alternative sources for FM and FO production does not substantially reduce the environmental impacts, largely due to their low conversion yields (Pelletier and Tyedmers, 2007).

## 3 Sustainability of fishmeal replacement with plant-based sources

A widely investigated strategy to enhance the sustainability of fish feed involves replacing animal-derived ingredients with plant-based alternatives. Aquaculture systems already rely on a broad range of plant ingredients, either as primary dietary components or as partial substitutes for FM and FO. These include grains such as wheat and corn, oilseeds such as soybean, sunflower, rapeseed, and cottonseed, and legumes including beans, lupins, and peas (Stickney and Gatlin, 2022). Among these, soybean meal (SM) is the most commonly used plant protein source in animal feeds worldwide (Hartman et al., 2011). Plant-based ingredients are generally widely available and more cost-effective than fishmeal (Chakraborty et al., 2019). Furthermore, crop traits, such as protein and oil content, can be improved through selective breeding and genetic engineering to better meet aquaculture nutritional requirements. Advances in genomics have elucidated the molecular mechanisms regulating key nutritional traits, providing breeders with tools to accelerate the development of crop varieties tailored for feed applications (Gatlin III et al., 2007).

Despite these advantages, the production of plant-based feed ingredients can substantially contribute to the environmental impact of aquaculture. Large areas of arable land are required to cultivate feed crops, potentially driving deforestation and increasing terrestrial ecotoxicity due to fertilizer use (Boissy et al., 2011). Additional concerns include the water footprint, particularly for irrigation (Mekonnen and Gerbens-Leenes, 2020), as well as energy consumption associated with cultivation, processing, and transport (Fréon et al., 2017).

Beyond environmental considerations, the nutritional and physiological implications of replacing FM and FO with plant-based ingredients must also be carefully evaluated (Aragão et al., 2022; Petereit et al., 2022; Glencross et al., 2024). Plant-derived ingredients often lack certain essential amino acids, such as taurine, which is particularly important for the growth and health of carnivorous fish species (Aragão et al., 2022). In addition, they may contain anti-nutritional factors that impair nutrient absorption and gut health. For example, soybean-derived saponins have been associated with enteritis in salmonids (Krogdahl et al., 2010). Diets rich in plant proteins may also compromise immune responses in fish, increasing disease susceptibility (Krogdahl et al., 2010; Aragão et al., 2022). To mitigate these effects, feeds are often supplemented with probiotics (live microorganisms that confer health benefits) and prebiotics (non-digestible compounds that stimulate the growth of beneficial gut microbiota). These additives can enhance intestinal health, improve immune responses, and strengthen disease resistance in farmed fish (Nawaz et al., 2018; Nimalan et al., 2022).

Among plant-based alternatives, microalgae have recently attracted growing interest (Olsen et al., 2024), particularly as potential substitutes for FM and FO in fish nutrition (Bandara, 2018; Sarker et al., 2020; Karapanagiotidis et al., 2022; Vázquez-Romero et al., 2024). Microalgae are rich in high-quality proteins, polyunsaturated fatty acids (including omega-6), vitamins, minerals, and antioxidant compounds. They also contain several bioactive molecules that can enhance growth performance and health in aquatic organisms (Dernekbasi et al., 2010; Roy and Pal, 2015). For example, the pigment astaxanthin, derived from the microalgae *Haematococcus pluvialis*, improves immune responses and mitigates oxidative stress in fish (Bandara, 2018; Lu et al., 2021; Patel et al., 2021). Microalgae are also promising prebiotic ingredients by promoting the proliferation of beneficial gut bacteria (Kononova et al., 2019; Patel et al., 2021). However, despite technological advancements and the exploration of alternative substrates such as wastewater to enhance productivity and reduce environmental burdens (Mennaa et al., 2015; Wuang et al., 2016; Mantovani et al., 2020; Geremia et al., 2021; Napolitano et al., 2022b; Pechsiri et al., 2023), large-scale algal production remains hampered by significant economic and environmental bottlenecks. These include high electricity demand, substantial water use, the need for nutrient-rich culture media, CO_2_ supply requirements, and energy-intensive harvesting processes (Han et al., 2019; Ragaza et al., 2020; Tzachor et al., 2022).

In recent years, increasing attention has been directed toward the valorization of agricultural and food industry by-products as alternative feed ingredients. The agricultural and food sectors generate substantial volumes of waste, usually disposed of on land or discharged into water bodies. Improving the sustainability of these sectors requires transforming such waste into value-added products (Freitas et al., 2021; Singh et al., 2021). Agro-industrial by-products can represent suitable aquafeed ingredients due to their relatively high content of proteins, carbohydrates, fatty acids, and bioactive compounds (Obirikorang et al., 2015; Pechsiri et al., 2023).

Within this context, BSG emerges as a particularly promising by-product for aquaculture applications (Karlsen and Skov, 2022). BSG has relatively high protein content (see below), low market cost, and consistent year-round availability. These properties make it a putative replacement for both animal-derived ingredients, such as fishmeal (Zerai et al., 2008), and plant-derived ingredients, such as soybean and wheat flour (Jayant et al., 2018). From a circular economy perspective, BSG offers significant potential as a sustainable food source in aquaculture (Kaur and Saxena, 2004; Jayant et al., 2018; Nazzaro et al., 2021; Karlsen and Skov, 2022). Its utilization could reduce dependence on conventional protein sources, mitigate associated environmental impacts, and contribute to the conservation of wild fish stocks. The following sections examine the advantages and limitations of using BSG as a replacement for more impactful ingredients.

## 4 Brewer’s spent grain as a replacement ingredient in aquafeed

### 4.1 Production of beer and related waste products

Global beer production has remained relatively stable over recent decades, reaching 1.82 billion hectoliters in 2022 (Statista, 2025). China is the leading producer, accounting for approximately 20% of global output, followed by the USA at 11% (Statista, 2025). Within the European Union, Member States produced 34.3 billion liters of alcoholic beer and nearly 1.8 billion liters of low- or non-alcoholic beer (Eurostat, 2024). BSG is the primary solid by-product of the brewing process (see Supplementary Figure S2 in Supplementary Material S1), representing approximately 80%–85% of the total waste generated by the industry (San Martin et al., 2020; Estévez et al., 2022; Karlsen and Skov, 2022). A major second by-product is brewer’s spent yeast (BSY), which accounts for around 10%-15% of total waste (San Martin et al., 2020; Estévez et al., 2022). Both BSG and BSY possess significant nutritional value and have attracted interest across multiple sectors (Mussatto et al., 2006; Wen et al., 2019). Additional brewery waste products, such as spent hop and trub, also represent potential sources of nutrients (e.g., proteins) and bioactive compounds (e.g., natural antioxidants) (Bravi et al., 2021; Gandolpho et al., 2025). Nowadays, in addition to limited disposal in landfills, BSG is predominantly utilized as a supplementary feed ingredient for livestock, especially cattle (Johnson et al., 2010). Beyond its application in aquaculture as a potential feed replacement ingredient, several potential uses have been proposed for BSG, including biogas production (Nganyira et al., 2023; 2025), incorporation into human food products (Schettino et al., 2021; Gutiérrez-Barrutia et al., 2022; Eche et al., 2025), and its use as a substrate for microbial cultivation and enzyme production (Casas-Godoy et al., 2023).

### 4.2 Brewer’s spent grain composition

BSG consists primarily of the husks, along with portions of barley’s pericarp and seed coat layers of barley (*Hordeum vulgare* L.), the principal raw material in beer production (Mussatto et al., 2006; Jay et al., 2008). BSG is a lignocellulosic material whose composition may vary depending on factors such as barley variety, brewing technology, and the use of adjuncts (Mussatto and Roberto, 2005; Robertson et al., 2010; Xiros and Christakopoulos, 2012; Lynch et al., 2015; Karlsen and Skov, 2022). The main components are non-starch polysaccharides (NSP, primarily cellulose and the hemicellulose arabinoxylan), lignin, proteins, lipids, and other minor polysaccharides (Xiros and Christakopoulos, 2012; Lynch et al., 2015; Karlsen and Skov, 2022; Zeko-Pivač et al., 2022). In addition, BSG also contains minerals and traces of vitamins (IAFFD, 2025), as well as significant concentrations of polyphenols and phenolic acids, compounds recognized for their antioxidant properties (Bravi et al., 2021).

**FIGURE 1:**
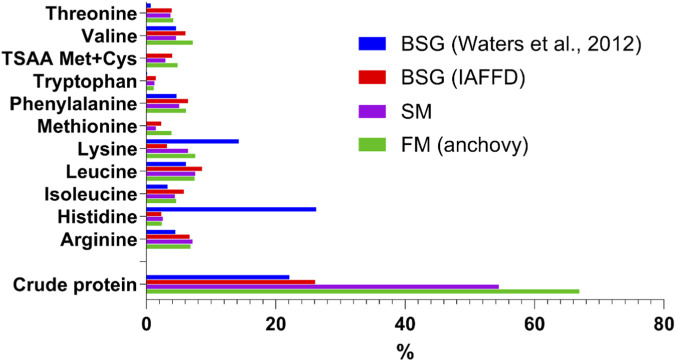
Comparison of the essential amino acid (EAAs) content of BSG, fish meal (FM), and soybean meal (SM). BSG data are from Waters et al. (2012) and from the IAFFD database (IAFFD, no date). EEA content is expressed as a percentage of crude protein (CP). CPs are in % dry mass. A detailed comparison with most of the ingredients used in aquafeed is reported in SM2.

On a dry-weight basis, non-starch polysaccharides (NSPs) account for more than 40% of BSG (Mussatto et al., 2006). Proteins and lignin are also present in substantial amounts, ranging from 15% to 27% and 11%–30%, respectively (Carvalheiro et al., 2004; Mussatto et al., 2006). Lipids constitute approximately 10% of the dry matter, starch ranges between 1-10%, and ash content is around 2% (Kanauchi et al., 2001; Mussatto and Roberto, 2005; Jay et al., 2008; Rojas-Chamorro et al., 2020). More than 50% of BSG proteins consist of hordeins (prolamin glycoproteins), which are the primary storage proteins in barley and are particularly rich in proline and glutamine (Celus et al., 2006; Lynch et al., 2015). The remaining protein fraction includes albumins, globulins, and glutelins (Lynch et al., 2015). Although the total protein content of BSG is lower than that of conventional aquafeed ingredients such as fishmeal (FM) and soybean meal (SM), its essential amino acids (EAAs) profile is comparatively balanced in relative terms (Figure 1 and in Supplementary Material S2). This characteristic enhances its potential value as a complementary protein source in aquafeed formulations.

### 4.3 Brewer’s spent grain replacement effects on growth performance parameters

Quite a few studies specifically examined the effects of BGS as a replacement ingredient for fishmeal on growth performance across fish species with different feeding habits (Table 1). The majority of these studies have focused on freshwater species. In most cases, dried and ground BSG was used to partially replace either fishmeal or plant-based ingredients in the diet. Some studies have evaluated processed forms of BSG, including enzyme-hydrolyzed BSG (He et al., 2020; Nazzaro et al., 2021) or fermented BSG (Estévez et al., 2022). Notably, He et al. (2020) utilized a protein-rich hydrolyzed BSG containing more than 45% CP. Similarly, the BSG used by Hassan et al. (2016), Jayant et al. (2018), and Chattaraj et al. (2024) was reported to contain more than 45% CP. It is not always clearly specified whether the BSG in these studies underwent processing prior to its inclusion in feeding trials. In one study, solid-state fermented BSG (BSG-SSF, see below) was evaluated in parallel with untreated BSG, allowing for a direct comparison of their effects (Estevão-Rodrigues et al., 2024).

**TABLE 1:** Effects of BSG introduction in aquafeed on growth performance and feed efficiency. The species tested are listed together with a synthesis of treatments and main effects.

Species	Treatment	Animal initial weight/Treatment duration	Effects	Literature source	Notes
Meagre (*Argyrosomus regius* Asso, 1801) (marine fish, carnivorous)	10% of BSG or BSG-SSF, reducing other plant ingredients	84 ± 1g/68 days	BSG, but not BSG-SSF, reduced growth performance and protein utilization efficiency of fish	Estevão-Rodrigues et al. (2025a)	^a^
Catla (*Catla catla* Hamilton 1822) (freshwater fish, zooplanktivore)	10%, 20%, 30%, and 40% of rice bran replaced by BSG	0.84 g/12 months	In 30% BSG, final body weight, SGR, and PER were higher than Control. FCR was lower in the 10% group	Kaur and Saxena (2004)	
Mrigal (*Cirrhina mrigala* (Hamilton 1822) (freshwater fish, phytoplanktivore)	10%, 20%, 30%, and 40% of rice bran replaced by BSG	3.89 g/12 months	No difference from control, except for an increase in FCR	Kaur and Saxena (2004)	
Reba carp *(Cirrhinus reba,* Hamilton 1822*)* (freshwater fish, planktivore)	25% of SM replaced by BSG	1.31 g/60 days	No difference from control in all growth performance parameters and digestibility	Chattaraj et al. (2024)	^b^
European seabass (*Dicentrarchus labrax* L.*)* (marine fish, carnivorous)	10% and 20% of BSG or BSG-SSF, reducing other plant ingredients	92 g/60 days	20% BSG-SSF significantly increased the digestibility of the diet	Estevão-Rodrigues et al. (2024)	^a^
	10% and 20% of BSG or BSG-SSF, reducing other plant ingredients	49g/10 weeks	Higher growth and feed efficiency than the control with 10% BSG-SSF, and lower with BSG (10% and 20%)	Estevão-Rodrigues et al. (2025b)	^a^
	Up to 0.4% crude lyophilized extracts of BSG-SSF	22g/15 days	Significant increase in feed and protein utilization	Fernandes et al. (2022)	
Channel catfish (*Ictalurus punctatus* Rafinesque, 1818) (freshwater fish, omnivorous)	27% and 55% BSG added to replace both soybean and wheat flour partially	5.2 ± 0.2 g/12 weeks	Lower growth compared to the control diet	Tidwell et al. (2023)	
Bata (*Labeo bata* Hamilton 1822) (freshwater fish, herbivore)	BSG replaces 100% of SM	3.32 ± 0.25 g/60 days	Survival rate and growth performance are not different from the control	Hassan et al. (2016)	^b^
Rohu *(Labeo rohita* Hamilton 1822) (potamodromous fish, omnivorous)	10%, 20%, 30%, and 40% BSG to replace rice bran	2.72g/12 months	The final body weight, SGR, and PER in group 30% were higher than in the control. FCR was lower in fish fed with 30% BSG	Kaur and Saxena (2004)	
Whiteleg shrimp *(Litopenaeus vannamei* Boone 1931) (marine shrimp, detritivorous, omnivorous)	10%, 30%, 50%, and 70% of fishmeal replaced by protein-rich H-BSG	1.10 ± 0.06 g/8 weeks	No difference with the control, except for the lower SFR and weight gain with 70% replacement	He et al. (2020)	^c^
Rainbow trout (*Oncorhynchus mykiss* Walbaum 1792) (freshwater fish, carnivorous)	20% BSG and H-BSG replacing fish meal in the diet	206.05 ± 41.18 g/30 days	BSG: no difference with control; H-BSG: significant reduction in growth; diet digestibility slightly reduced	Nazzaro et al. (2021)	^d^
	7.5% and 15% BSG or H-BSG in diets	77.90 ± 8.97 g/60 days	No difference from the control diet.	Estévez et al. (2022)	^d^
Nile tilapia (*Oreochromis niloticus* L.) (freshwater fish, herbivore)	25%, 50%, 75%, and 100% of fishmeal replaced by BSG	24.8 6 5.0 g/10 weeks	No significant difference in body weight gain between the control diet and diets that replaced 25% or 50% of fishmeal with BSG. No significant differences in FCR between the control and 25%, 50% or 75% BSG diets. PER was the highest in the control group and the lowest in the 100% BSG group	Zerai et al. (2008)	
	27% and 55% BSG added to replace both soybean and wheat flour partially	5.0 ± 1.2 g/8 weeks	Lower growth compared to the control diet	Tidwell et al. (2023)	
Striped catfish (*Pangasianodon hypophthalmus* Sauvage, 1878) (freshwater fish, omnivorous)	25%, 50%, 75%, and 100% of soybean replaced by BreweryWaste1	2.2 ± 0.02 g/60 days	Most parameters, including SGR and FCR, were not affected by 25% and 50% BSG replacement and were reduced by higher BSG levels	Jayant et al. (2018)	^b^
Gilthead seabream (*Sparus aurata* L.) (marine fish, carnivorous)	7.5%, 15% and 20% BSG; 20% H-BSG in the diets. Other plant meals were accordingly modified to keep diets isonitrogenous	94.49 ± 9.07 g/70 days	20% BSG or H-BSG lowered both growth and feed utilization	Estévez et al. (2021)	
	20% BSG, H-BSG, or MW-BSG	60.49 ± 0.26g/3 months	MW-BSG improved growth and feed efficiency	Martínez-Antequera, et al. (2025)	
	20% D-BSG and H-BSG replacing fish meal in the diet	253.01 ± 27.68 g/30 days	No difference with the control diet; diet digestibility slightly reduced	Nazzaro et al. (2021)	^d^

Initial weights are as mean ± SD, when provided by the authors.

BSG: brewer spent grain; H-BSG: hydrolyzed BSG; MW-BSG: microwave heat pretreated BSG; BSG-SSF: solid-state fermented BSG; SGR: specific growth rate; FCR: feed conversion ratio; PER: protein efficiency ratio.

^a^ BSG-SSF is produced by solid-state fermentation of BSG with *Aspergillus ibericus*; compared with BSG, it contains more proteins (32% vs. 27%) and less cellulose (15% vs. 21%).

^b^ Brewery waste: the “brewery spent grain” used in these studies was rich in CPs, 47.26% dry mass.

^c^ BSG, was hydrolyzed and concentrated, so the CP content was about 46%.

^d^ BSG and H-BSG had a similar CP content (25% and 22%, respectively).

Despite the heterogeneity of experimental designs and feeding protocols, some general conclusions can be drawn. Overall, most of these studies suggest that a partial but significant replacement of fish or vegetal protein sources with BSG does not negatively affect growth performance and, in some cases, may even improve it. However, the effectiveness of BSG replacement likely appears to depend on several factors, including fish species, dietary inclusion level, and the type of BSG processing applied. For example, studies on iridescent striped catfish (*Pangasianodon hypophthalmus* Sauvage, 1878) and Nile tilapia (*Oreochromis niloticus* L.) reported that diets in which up to 50% of fishmeal or soybean meal was replaced with BSG resulted in comparable or even higher weight gain and specific growth rate (SGR) relative to control groups (Zerai et al., 2008; Jayant et al., 2018). Studies carried out on gilthead seabream (*Sparus aurata* Linnaeus, 1758), rainbow trout (*Oncorhynchus mykiss* Walbaum, 1792), and Nile tilapia (*O. niloticus*) have shown that the BSG-containing diets performed comparably to control diets, without affecting final weight, relative growth rate (RGR), or feed conversion ratio (FCR, the ratio between the total weight of feed fed to animals and the harvested animals) (Zerai et al., 2008; Nazzaro et al., 2021; Estévez et al., 2022). However, when BSG replaces more than 50% of fishmeal, growth and performance can be negatively affected compared to traditional feeds. For instance, in trials on Pacific white shrimp (*Litopenaeus vannamei* Boone 1931), a 70% fishmeal replacement with BSG resulted in lower specific growth rates (SGR) compared to the control diet (He et al., 2020). Only one study on Nile tilapia reported reduced growth rate at both BSG concentrations tested (27% and 55%) (Tidwell et al., 2023).

The use of processed BSG derivatives, such as solid-state fermented BSG (BSG-SSF) or BSG-derived enzyme extracts, generally enhances feed efficiency and growth performance in fish. For instance, inclusion of 10% BSG-SSF improved both growth and feed efficiency in *Argyrosomus regius* and *D. labrax* (Estevão-Rodrigues et al., 2025a; Estevão-Rodrigues et al., 2025b). Similarly, crude extracts from fermented BSG, incorporated at 0.4% into a plant-based diet, enhanced digestibility as well as feed and protein efficiency ratios in *D. labrax* (Fernandes et al., 2021; 2022). Additionally, microwave heat–treated BSG (MW-BSG), included at 20% of the diet, improved growth and feed efficiency in gilthead seabream (*S. auratus*) (Martínez-Antequera et al., 2025).

In summary, although a comprehensive understanding of BSG inclusion in aquafeed is still developing, the available evidence strongly indicates that replacing up to approximately 50% of conventional protein sources with BSG can achieve growth performance comparable to control diets. This observation highlights the potential of BSG as a viable and sustainable protein source in aquafeed, which can be used as a partial replacement for FM, soybean, or both in aquaculture feeds.

## 5 Brewer’s spent grain effects on redox homeostasis and health status of fish

Oxidative stress is a critical concern in aquaculture, driving interest in dietary strategies to enhance the antioxidant capacity and overall health of cultured species (Ciji and Akhtar, 2021; He et al., 2023; Song et al., 2023). Beyond its role as a protein source in animal feeding, BSG is recognized for its antioxidant properties, which are primarily attributed to its high content of phenolic compounds—a group of bioactive molecules that can prevent oxidative damage under conditions of excessive cellular production of Reactive Oxygen Species (ROS) (Bravi et al., 2021). Demonstrating that BSG can enhance the oxidative defense mechanisms in fish would provide a compelling additional rationale for its inclusion as a functional ingredient in aquafeeds.

### 5.1 Oxidative stress in aquaculture

ROS are molecules produced by multiple cellular pathways, with mitochondria serving as both a primary source and a major target (Napolitano et al., 2021). While ROS play several essential roles in maintaining normal cellular functionality (Napolitano et al., 2022a), their high reactivity also makes them capable of oxidatively damaging lipids, proteins, and nucleic acids, thereby impairing cell integrity and functionality (Napolitano et al., 2022a). To mitigate ROS-induced damage and maintain redox homeostasis, cells rely on an antioxidant system that efficiently preserves redox homeostasis (Napolitano et al., 2023; Subba et al., 2024). When ROS levels exceed the capacity of this system, oxidative stress occurs, which can compromise cellular function and increase the organism’s susceptibility to disease (Napolitano et al., 2023; Subba et al., 2024).

In aquaculture, organisms can be chronically exposed to stress conditions, including poor water quality, pollution, improper management practices such as high stocking densities, and inadequate nutrition (Jeney, 2017; Hardy and Kaushik, 2021). These conditions increase susceptibility to pathogens and compromise overall fish health and wellbeing (Noga, 2010). Environmental stress conditions are often associated with oxidative stress resulting from disrupted mitochondrial function, increased ROS production, and a reduction in the antioxidant defenses (Chowdhury and Saikia, 2020; Jia et al., 2022; Shi et al., 2022; Song et al., 2023). Excessive or unbalanced ROS levels can impair gill respiratory efficiency (Lushchak, 2011; Lushchak, 2016), induce liver dysfunction (Atli et al., 2006), and interfere with nutrient absorption in the intestine (Lushchak, 2016). Oxidative stress also affects other organs, such as muscles, brain (Costantini et al., 2010), and kidneys (Lushchak, 2011), while the heart, gonads, skin, and fins may also be affected, although their involvement in the oxidative stress response is less commonly reported.

The severity and specific effects of oxidative stress depend on multiple factors, including the type, intensity, and duration of the stressors, as well as the species, age, and developmental stage of the fish. Moreover, the oxidative stress response may vary across different tissues (Osório et al., 2022; Subaramaniyam et al., 2023). A thorough understanding of the underlying mechanisms and tissue-specific impacts of oxidative stress is essential for designing effective strategies to mitigate its effects and improve resilience in aquaculture systems (Zhang et al., 2022).

Supplementing fish feed with antioxidant compounds is currently the primary approach used to mitigate oxidative stress in aquaculture. Synthetic antioxidants, such as ethoxyquin, butylated hydroxyanisole (BHA), and butylated hydroxytoluene (BHT), are among the most widely used additives in food and feed formulations (Lundebye et al., 2010; Błaszczyk et al., 2013; Kandeel et al., 2024). More recently, there has been growing interest in incorporating natural antioxidants into fish feeds, as these compounds can enhance growth and product quality while promoting the sustainability of aquaculture systems (Tsiplakou et al., 2021; Pereira et al., 2022; Hu et al., 2025).

### 5.2 Antioxidant properties of brewer’s spent grain

Natural sources of polyphenols have been proposed as a viable alternative to synthetic antioxidants for promoting fish health and enhancing aquaculture productivity (Ahmadifar et al., 2021). The potential of BSG as a dietary antioxidant supplement has, to date, been primarily assessed in non-fish models. Polyphenols and phenolic acids in BSG may exert anti-inflammatory and antioxidant effects either by directly neutralizing reactive oxygen species (Oliva et al., 2023) or by indirectly enhancing endogenous antioxidant defenses (Fernandes et al., 2022). For example, McCarthy et al. (2014) reported that phenolic extracts from BSG increased cellular antioxidant capacity and reduced the production of pro-inflammatory interleukins in U87 cells. Similarly, Merten et al. (2022) observed a significant, dose-dependent beneficial effect of BSG as a functional food in a cell model of the small intestine, which was correlated with the content of phenolic compounds and their antioxidant activity. *In vivo* studies have also been conducted; for instance, Boontiam et al. (2022) conducted a 28-day feeding trial in weaning pigs, incorporating diets with 10% and 20% BSG fortified with 0.1% multienzymes. Dietary inclusion of BSG has been shown to enhance total antioxidant capacity (TAC) and stimulate the activity of the antioxidant enzyme superoxide dismutase (SOD). At the same time, levels of malondialdehyde (MDA), a widely used biomarker of advanced lipid oxidative damage, were reduced in animals receiving 20% BSG. These findings are consistent with earlier observations on broiler chicken (Ashour et al., 2019), where graded levels of dietary BSG (3%, 6%, 9%, and 12%) resulted in a dose-dependent increase in glutathione peroxidase (GPx) activity and a parallel decrease in MDA concentrations. In the same study, reductions in serum aspartate aminotransferase activity, urea, and creatinine were also reported, suggesting potential improvements in liver and kidney function. BSG supplementation also protects against a high-fat diet in rats by reducing the amount of triglycerides and LDL (Low-Density Lipoprotein) in plasma (Orzel et al., 2018). Owing to its high content of dietary fiber, proteins, and bioactive phenolic compounds, BSG has been recognized as a valuable antioxidant-rich food ingredient for human consumption (Ikram et al., 2017; Schettino et al., 2021), which can potentially help prevent various diseases. Polyphenols have also been implicated in the anti-inflammatory effects of BSG protein extract and its associated hydrolysates (McCarthy et al., 2013). Information on the antioxidant effects of BSG in fish nutrition remains limited. In a study conducted by Fernandes et al. (2022) on juvenile European sea bass (*D. labrax* L.), a BSG extract enriched in carbohydrases and antioxidants (obtained through solid-state fermentation, SSF), a process in which BSG serves as a substrate for microorganisms such as *Aspergillus* spp. or *Bacillus* spp.) significantly reduced lipid peroxidation (LPO) in the intestine and muscle. Additionally, the extract enhanced the activity of antioxidant enzymes in both the intestine and liver. These findings were partially confirmed by Estevão-Rodrigues et al. (2025b), further supporting the functional potential of processed BSG derivatives. Overall, although the available evidence is still limited, current findings indicate that BSG has excellent potential as a natural antioxidant source. This potential warrants further investigation into its application as a functional antioxidant ingredient in aquafeeds.

### 5.3 Immunomodulatory properties of Brewer’s spent grain

Moderate inclusion of brewers’ spent grain (BSG) in mammalian diets has been shown to exert immunomodulatory effects, particularly when BSG is processed into extracts or protein hydrolysates. Enzymatically hydrolyzed BSG has been reported to stimulate the production of both pro- and anti-inflammatory cytokines in rat immune cells (Cian et al., 2020; Barrera-León et al., 2025), suggesting a modulatory rather than purely stimulatory immune response. Similarly, extracts obtained from pulsed electric field (PEF)-treated BSG have been shown to induce the expression of pro-inflammatory cytokines, including IL-1β, IL-6, and TNF-α, as well as chemokines such as IL-8, MCP-1, and MIP-1α in human immune cell assays (Kumari et al., 2019).

The antioxidant properties of BSG are closely associated with its immunomodulatory activity. Phenolic-enriched BSG extracts have been shown to increase the activity of key antioxidant defenses, including superoxide dismutase (SOD), catalase, and glutathione (GSH), while simultaneously decreasing the production of cytokines such as IL-2, IL-4, IL-10, and IFN-γ in stimulated T cells. This pattern suggests coordinated antioxidant and anti-inflammatory responses (McCarthy et al., 2014). Moreover, BSG-derived protein hydrolysates and bioactive peptides exhibit both immunoregulatory and antioxidant effects. *In vitro* studies have demonstrated their capacity to protect against oxidative damage (Connolly et al., 2013; McCarthy et al., 2014) and to reduce IL-6 production in lipopolysaccharide (LPS)-stimulated macrophages (Connolly et al., 2013). *In vivo* evidence further indicates that BSG supplementation exerts combined antioxidant and anti-inflammatory effects along the liver–spleen axis in sucrose-fed rats (Oliva et al., 2023). It has been proposed that the enhancement of immune cell function following BSG supplementation may be attributable to an improved cellular redox state, resulting from its antioxidant capacity (Kaur and Saxena, 2004; Wen et al., 2019). However, most available studies have merely reported the co-occurrence of antioxidant and immunomodulatory effects, and a direct mechanistic link between these responses has yet to be clearly established.

Processed forms of BSG have also demonstrated immunomodulatory activity in fish. For example, dietary inclusion of enzymatically or microwave-pretreated BSG at 20% has been shown to modulate gut microbiota composition and increase intestinal alkaline phosphatase activity in gilthead seabream (*S. aurata*) (Martínez-Antequera et al., 2025). Furthermore, a BSG extract (BEP), produced through cellulase and hemicellulase hydrolysis, upregulated the expression of pro-inflammatory cytokines and antioxidant enzymes (including SOD, GPx, and glutathione reductase (GR) in a rainbow trout intestinal epithelial cell line (RTgutGC) (Cárcamo et al., 2026). Although several studies have documented pronounced immunomodulatory effects of brewers’ spent yeast (BSY) in fish nutrition, the immunological impact of untreated BSG in fish diets remains largely unexplored.

### 5.4 Other effects of brewer’s spent grain on fish health status

Several studies have assessed the metabolic and physiological status of fish fed diets containing BSG. In juvenile European seabass (*D. labrax*), both untreated BSG and solid-state fermented BSG (BSG-SSF) reduced plasma glucose and phospholipid levels, as well as hepatic glucokinase and malic enzyme activities, indicating a shift in carbohydrate and lipid metabolism (Estevão-Rodrigues et al., 2025a). In gilthead seabream (*S. aurata*), inclusion of pretreated BSG altered plasma metabolic markers, decreased lactate concentrations, and increased alkaline phosphatase activity in skin mucus, suggesting modulation of both metabolic pathways and mucosal immune responses (Martínez-Antequera et al., 2025). Replacement of soybean meal or fishmeal with BSG did not appear to compromise physiological status in other species. In striped catfish, inclusion levels of up to 70% BSG (replacing soybean meal) did not affect protein retention or body composition (Jayant et al., 2018). Similarly, in reba carp, a 13% substitution of soybean meal with BSG did not alter body composition or serum alanine aminotransferase (ALT) and aspartate aminotransferase (AST) activities, which are indicators of liver function (Chattaraj et al., 2024). Overall, these findings suggest that partial replacement of less sustainable protein sources with BSG generally does not impair fish health, although outcomes depend on species and inclusion level. Moreover, processed or pretreated BSG may even enhance certain aspects of metabolic and physiological status.

## 6 Main bottlenecks of applying brewer's spent grain in aquafeed

Despite its reported and potential benefits in aquafeeds, the use of BSG also presents certain limitations. As with many protein sources of plant origin, BSG may pose challenges related to lower digestibility and palatability compared to fish meal, largely due to its high content of antinutritional factors (ANFs). Moreover, its lower protein content compared to traditional ingredients (e.g., FM or SM) may increase the risk of deficiencies in specific essential amino acids (AAs) if diets are not properly balanced (Kokou and Fountoulaki, 2018; Karlsen and Skov, 2022).

### 6.1 Antinutritional factors, digestibility, and palatability

Plant ANFs include lectins, phytic acid, alkaloids, protease inhibitors, tannins, and indigestible non-starch polysaccharides (NSPs). As in most animals, fish lack the endogenous enzymes required to hydrolyze the β-glycosidic bonds in NSPs. Thus, plant-based protein sources are often less digestible and may influence intestinal microflora, impair digestion, reduce nutrient absorption, and negatively affect fish’s intestinal health, immune modulation, and growth (Krogdahl et al., 2010; Kokou and Fountoulaki, 2018; Hardy and Kaushik, 2021; Karlsen and Skov, 2022). Other anti-nutritional factors in plant-based fish feed ingredients include protease inhibitors, lectins, tannins, saponins, phytic acid, glucosinolates, and some alkaloids (see Supplementary Material S2 for a detailed list and content).

Unrefined BSG nutritional value in aquaculture is affected by its high content of ANFs, which mainly include NSPs (cellulose, hemicellulose, β-glucan, and pectins) and lignin (He et al., 2020). Tannins and phytic acid are also present, although in comparatively lower concentrations. Notably, BSG contains higher lignin and fiber concentrations than SM (Karlsen and Skov, 2022). The ANFs in BSG may affect feed intake, efficiency, and fish health (Karlsen and Skov, 2022). Recently, a study on Nile tilapia fed a diet containing 20% BSG reported a crude protein digestibility of 67% while energy digestibility was reduced, likely due to the high fiber content (Glowka et al., 2025).

One strategy to overcome the low digestibility of BSG could be to reduce the indigestible components (Martínez-Antequera et al., 2025). For example, lignin and fiber in BSG can be reduced to negligible quantities through refinement processes such as chemical fractionation (protein extraction and removal of fiber and lignin) or through the conversion of lignocellulose fractions into more digestible components using biological, chemical, or enzymatic treatments (He et al., 2020; Karlsen and Skov, 2022). Solid-state fermentation of BSG, which promotes the hydrolysis of NSPs, has been shown to improve diet digestibility in juvenile *D. labrax* (Estevão-Rodrigues et al., 2024). On the other hand, only a slight reduction in the apparent digestibility of diets was observed by Nazzaro et al. (2021) when 20% BSG or hydrolyzed BSG was used to replace fishmeal, suggesting that moderate inclusion levels—particularly when combined with processing—may mitigate negative effects on nutrient utilization.

Another issue regarding BSG inclusion in fish diets is feed palatability. Several studies have found that high BSG inclusion can reduce feed intake in fish, suggesting lower palatability, which may be related to the increased proportion of plant-based proteins (Oliva-Teles and Gonçalves, 2001; Estévez et al., 2021; 2022). Nevertheless, additional research is needed to comprehensively evaluate the factors influencing BSG palatability and its acceptability across different fish species.

### 6.2 Brewer’s spent grain proteins as a source of essential amino acids

Proteins and AAs are the most critical components for animals, as they underpin key physiological and biochemical processes as well as tissue growth. However, animals (including aquatic animals such as fish and shrimp) are unable to synthesize all AAs required for protein turnover, the mechanism through which tissues are renewed, and physiological wellbeing is maintained. Consequently, these organisms must acquire essential AAs (EAAs) through dietary protein intake (Hardy and Kaushik, 2021). The requirement for EAAs is species-specific, making it crucial to provide diets containing adequate levels of both total proteins and individual AAs (Hardy and Kaushik, 2021). Generally, for most fish, a 1:1 ratio between total EAAs and total non-essential AAs (NEAAs) in dietary protein is considered optimal for efficient body protein synthesis (Hardy and Kaushik, 2021). The EAAs required by fish include arginine, histidine, isoleucine, leucine, valine, lysine, sulfur amino acids (methionine + cysteine), aromatic amino acids (phenylalanine + tyrosine), threonine, and tryptophan. According to a recent meta-analysis (Xing et al., 2024), the dietary requirements for these EAAs, expressed as a percentage of CPs, range from 0.9% for tryptophan to 6.2% for aromatic amino acids.

Alternative protein sources, such as plant-based ingredients or food industry by-products, fail to provide a complete or well-balanced amino acid profile when compared with fish meal, which is naturally rich in EAAs. Most plant protein sources used in aquaculture feeds, such as corn, wheat, and barley, tend to be deficient in key EAAs, including lysine, methionine, arginine, and histidine (see Supplementary Material S2) (He et al., 2019; He et al., 2020; Karlsen and Skov, 2022).

Concerning the content of EAAs in BSG, Waters et al. (2012) reported very high levels of EAAs, notably lysine (14.3% of CP) and leucine (6.12% of CP). However, these elevations have not been confirmed by subsequent studies (Connolly et al., 2013; Wen et al., 2019; Alonso-Riaño et al., 2021). Overall, the EAAs’ content of BSG, when expressed as a percentage of CP, can be considered comparable to that of most ingredients currently used in fish feeds (see Supplementary Material S2 for details), indicating that the total content of crude proteins level is the primary limiting factor. Interestingly, BSG waste from the craft beer industry has been reported to contain a high proportion of free amino acids, approximately 40% of which are EAAs, compared with BSG waste generated by large multinational beer industry (Jin et al., 2022). Consistently, BSG supplied by a local microbrewing company was reported to contain a higher protein content (32%) than that reported in most other studies evaluating BSG as a fish meal replacement (Zerai et al., 2008).

Beyond formulating diets by combining protein sources with different amino acid profiles, it is possible to correct the AA deficiency in a BSG-based diet by using two main approaches:

- Increasing the total protein percentage of BSG. Post-processing treatments can substantially enhance the content of total proteins of BSG. For instance, alkaline treatment with sodium hydroxide followed by enzymatic hydrolysis using alcalase produces a high-protein ingredient that has been shown to replace up to 50% of fish meal in shrimp diets without adversely affecting specific growth rate (SGR) or feed conversion ratio (FCR) (He et al., 2019; 2020). Similarly, solid-state fermentation (SSF) of BSG, which, as mentioned above, may improve digestibility and antioxidant content, also increases total CP levels, thereby enhancing the overall nutritional profile of BSG (Estevão-Rodrigues et al., 2024). In European seabass (*D. labrax*), dietary inclusion of BSG-SSF at 10% improved growth performance and feed efficiency, while inclusion at 20% resulted in performance comparable to that of the control diet (Estevão-Rodrigues et al., 2025b).
- Supplementation with crystalline amino acids (CAAs). Among the widespread successes of biotechnological innovation is the substantial reduction in the cost of large-scale AAs production for use in animal feeds (National Research Council, Division on Earth and Life Studies, Board on Agriculture and Natural Resources, Committee on the Nutrient Requirements of Fish and Shrimp, 2011). Amino acids such as DL-methionine and L-Lysine are now routinely produced through chemical synthesis or microbial fermentation. Given the high cost of fishmeal and the cheapness of plant protein source with “imperfect” EAA profiles, CCAs represent a potentially effective strategy to meet the EAA requirements of fish, thereby enabling the formulation of nutritionally adequate and low-cost aquafeeds (National Research Council, Division on Earth and Life Studies, Board on Agriculture and Natural Resources, Committee on the Nutrient Requirements of Fish and Shrimp, 2011; Hardy and Kaushik, 2021; Karlsen and Skov, 2022). CAAs have been successfully used in commercial feeds to meet the EAA needs of land animals for terrestrial livestock for more than 40–60 years (National Research Council, Division on Earth and Life Studies, Board on Agriculture and Natural Resources, Committee on the Nutrient Requirements of Fish and Shrimp, 2011; Hardy and Kaushik, 2021). However, despite their economic relevance in feed formulations, their widespread adoption in aquaculture has been slow, largely due to uncertainties regarding their utilization efficiency in aquatic species (National Research Council, Division on Earth and Life Studies, Board on Agriculture and Natural Resources, Committee on the Nutrient Requirements of Fish and Shrimp, 2011). While some studies have demonstrated that CAAs are utilized as efficiently as EAAs derived from intact dietary proteins (Williams et al., 2001; Rollin et al., 2003), others have reported reduced efficiency (Peres and Oliva-Teles, 2005; Dabrowski et al., 2010).

Both approaches appear promising. However, further research is required to comprehensively assess the global environmental sustainability of SSF-treated BSG and CAA-enriched BSG–based diets. Such evaluations should adopt a holistic perspective and explicitly account for the environmental impacts associated with crystalline amino acid synthesis as well as BSG processing and post-treatment steps.

## 7 Environmental impacts of using BSG for fish feeding in aquaculture

Despite the limitations described above, we can infer from current knowledge that BSG can partially replace traditional fish feed ingredients without negatively affecting fish growth and, depending on species and inclusion level, may even enhance animal wellbeing. However, what about the consequences of using BSG in fish feed in terms of environmental impact assessment?

At present, there is a clear paucity of studies specifically addressing the environmental sustainability of BSG use in aquafeed. To complement the overview of biological and applicative aspects of BSG supplementation with an environmental perspective, this review includes the results of a life cycle assessment (LCA) analysis. In this analysis, the potential environmental impacts of two hypothetical fish diets incorporating BSG, partially replacing ingredients such as FM, wheat flour, and SM, were evaluated (see Supplementary Material S1 for details). LCA, as standardized by the International Organization for Standardization (ISO, 2006), is a key methodological tool for assessing environmental sustainability in aquaculture production systems (Cao et al., 2013; Bohnes and Laurent, 2019). Our analysis focused on a diet for *O. niloticus*, one of the most widely farmed fish species in global aquaculture, with production exceeding 5 million tons in 2022 (FAO, 2024). This species is an omnivorous species and capable of efficiently digesting plant-derived ingredients (Webster and Lim, 2002; FAO, 2024). Diet composition for *O. niloticus* can vary considerably depending on life stage (FAO, 2025). For the present assessment, we adopted the reference diet proposed by Zerai et al. (2008), in which the main ingredients were wheat flour (44%), FM (25%), and SM (25%). Using AFOS (Animal Feed Formulation Software, www.animalfeedsoftware.com), we formulated three nutritionally balanced diets: a control diet according to Zerai et al. (2008), a D1 diet containing 10% BSG, and a D2 diet containing 37% BSG (see Supplementary Tables S1, S2 for details on formulation and composition, Supplementary Material S1). The inclusion of BSG required a reduction in wheat meal (by 15% and 25% in D1 and D2, respectively) and fish meal (by 5% and 8% in D1 and D2, respectively), corresponding to an overall reduction in fish meal usage of 20% in D1 and 32% in D2. It is worth noting that to maintain nutritional balance, the SM content had to be increased in D1, while in D2, both FM and SM were reduced.

We evaluated the Fish-In to Fish-Out (FIFO) ratio for the three experimental diets and applied LCA methodology to each of them (see Supplementary Material S1 for details). The FIFO ratio quantifies the amount of wild-caught fish required to produce 1 kg of farmed fish and is widely used as a simplified benchmark to assess the impact of FM and FO used in aquaculture on wild fish stocks (Glencross et al., 2024). Although the FIFO metric has recognized limitations, such as its inability to distinguish between fisheries-derived ingredients and processing by-products (Kok et al., 2020; Aas et al., 2022; IFFO, 2025), remains a commonly adopted indicator of the sector’s progress toward improved environmental performance. FIFO values are influenced by the amount of feed required to achieve 1 kg of biomass gain, as expressed by the feed conversion ratio (FCR) (El-Sayed, 2013; Kok et al., 2020). At the global level, the use of FM and FO in aquaculture declined steadily between 2000 and 2020, with the overall FIFO value dropping from 0.66 to 0.27 (IFFO, no date). In our analysis, the inclusion of BSG in the diets resulted in a marked reduction in FIFO values, calculated following Glencross et al. (2024), of 18% for D1 and 31% for D2 (see Supplementary Material S1 for details). These results indicate that even a relatively modest reduction in fish meal inclusion (5%–8%) can substantially lower pressure on wild fish stocks, highlighting the potential environmental benefits of incorporating BSG into aquafeeds.

The LCA results for the production of the three diets (Supplementary Figures S3–S5 in Supplementary Material S1) indicate that the environmental impacts are primarily driven by the inclusion levels of FM, SM, and wheat flour. In most environmental impact categories, SM and wheat flour contribute the largest shares. However, soybean cultivation may reduce impacts in the human non-carcinogenic toxicity category, likely due to the plant’s capacity to uptake heavy metals such as nickel and zinc. Fish meal shows the highest contributions to Ozone formation, Human health, Ozone terrestrial ecosystem, Terrestrial ecotoxicity, Marine ecotoxicity, and Fossil resource scarcity categories. The D2 diet (Supplementary Figure S5 in Supplementary Material S1), characterized by the highest inclusion of BSG, exhibits the lowest overall environmental impact. This finding suggests that incorporating BSG as an alternative ingredient can effectively reduce the environmental burdens associated with aquafeed production. Conversely, the D1 diet (Supplementary Figure S4 in Supplementary Material S1), although reducing the impacts associated with fish meal through increased soybean inclusion, results in higher contributions to several impact categories such as Global warming, Freshwater eutrophication, Freshwater ecotoxicity, Human carcinogenic, Land use, Mineral resource scarcity, and Water consumption. Consequently, when comparing the environmental sustainability of the three diets (Supplementary Figure S6 in Supplementary Material S1), the Control and D1 diets displayed similar impacts. Overall, the comparison highlights the importance of carefully balancing ingredient substitution strategies, as reducing reliance on fish meal does not necessarily translate into lower environmental impacts across all categories.

Although the D2 diet shows the lowest contribution across all environmental impact categories, it may not necessarily represent the optimal formulation for *O. niloticus*, given its high BSG inclusion level. For instance, Tidwell et al. (2023) reported that replacing with BSG reduced growth performance in this species when BSG inclusion exceeded 27%.

Overall, the analysis suggests that substituting fish meal, wheat flour, and soybean meal with BSG can alleviate pressure on fish stocks and reduce environmental impacts. However, such benefits are contingent upon maintaining neutral or positive effects on growth performance, as indicated by several studies to date.

## 8 Conclusion

Improving the sustainability of aquaculture, the fastest-growing food production sector, offers multiple environmental, social, and economic benefits. In line with the principles of the Blue Economy (Bennett et al., 2019) and several of the Sustainable Development Goals (SDGs) outlined in the UN 2030 Agenda (Walsh et al., 2022), sustainable aquaculture can contribute to the conservation and responsible use of marine resources. Moreover, it can support the achievement of the SDGs “No poverty” and “Zero hunger” by helping to reduce poverty and providing food for people. Finally, promoting sustainable aquaculture practices can advance the goals of “Good Health and Wellbeing” and “Climate Action,” generating positive outcomes for both human health and climate mitigation.

Within a circular economy framework and from an environmental perspective, replacing traditional feed ingredients, including plant-based ones, with BSG in aquaculture diets has the potential to reduce the sector’s contribution to major environmental challenges, such as global warming and resource scarcity (e.g., land and water). Our review suggests that the partial substitution of more impactful ingredients, such as fish meal or SM, with BSG may represent an environmentally sound strategy for aquafeed formulation. However, the evidence also highlights the need for a cautious and balanced approach when incorporating BSG into aquaculture feeds. An assessment that integrates environmental, nutritional, economic, and zootechnical perspectives is still required. While several studies report that BSG inclusion can maintain satisfactory growth performance, at least in certain species and at moderate inclusion levels, some findings also suggest potential benefits for animal welfare.

However, the high levels of lignin and non-starch polysaccharides (NSPs) in BSG, often regarded as antinutritional factors, especially for carnivorous species, may affect diet digestibility. In addition, its crude protein content is not comparable to that of fish meal and may be inadequate to fully meet the essential amino acid requirements of farmed fish. These nutritional constraints currently limit the broader adoption of BSG in aquafeed formulations. On the other hand, the nutritional quality of BSG can be enhanced through various processing techniques aimed at reducing ANFs and increasing protein content. Further research is required before such approaches can be reliably implemented at scale in aquafeed production. Moreover, comprehensive assessments of both overall and category-specific environmental impacts—covering all stages of feed production—are essential to accurately quantify and optimize BSG substitution strategies. Such integrated evaluations would support the development of replacement protocols consistent with the broader goal of enhancing the sustainability of the aquaculture sector.

## References

[B1] Aas T. S. Åsgård T. Ytrestøyl T. (2022). Utilization of feed resources in the production of Atlantic salmon (*Salmo salar*) in Norway: an update for 2020. Aquac. Rep. 26, 101316. 10.1016/j.aqrep.2022.101316

[B2] Ahmad A. L. Chin J. Y. Mohd Harun M. H. Z. Low S. C. (2022). Environmental impacts and imperative technologies towards sustainable treatment of aquaculture wastewater: a review. J. Water Process Eng. 46, 102553. 10.1016/j.jwpe.2021.102553

[B3] Ahmadifar E. Yousefi M. Karimi M. Raieni R. F. Dadar M. Yilmaz S. (2021). Benefits of dietary polyphenols and polyphenol-rich additives to aquatic animal health: an overview. Rev. Fish. Sci. and Aquac. 29, 478–511. 10.1080/23308249.2020.1818689

[B4] Alonso-Riaño P. Sanz M. T. Benito-Román O. Beltrán S. Trigueros E. (2021). Subcritical water as hydrolytic medium to recover and fractionate the protein fraction and phenolic compounds from craft brewer’s spent grain. Food Chem. 351, 129264. 10.1016/j.foodchem.2021.129264 33662908

[B5] Aragão C. Gonçalves A. T. Costas B. Azeredo R. Xavier M. J. Engrola S. (2022). Alternative proteins for fish diets: implications beyond growth. Animals 12, 1211. 10.3390/ani12091211 35565636 PMC9103129

[B6] Ashour E. A. El-Hack M. E. A. El-Hindawy M. M. Attia A. I. Osman A. O. Swelum A. A. (2019). Impacts of dietary inclusion of dried brewers’ grains on growth, carcass traits, meat quality, nutrient digestibility and blood biochemical indices of broilers. S. Afr. J. Anim. Sci. 49, 573–584. 10.4314/sajas.v49i3.18

[B7] Atli G. Alptekin Ö. Tükel S. Canli M. (2006). Response of catalase activity to Ag^+^, Cd^2+^, Cr^6+^, Cu^2+^ and Zn^2+^ in five tissues of freshwater fish *Oreochromis niloticus* . Comp. Biochem. Physiol. C. Toxicol. Pharmacol. 143, 218–224. 10.1016/j.cbpc.2006.02.003 16581305

[B8] Bandara T. (2018). Alternative feed ingredients in aquaculture: opportunities and challenges. J. Entomol. Zool. Stud. 6, 3087–3094. Available online at: https://www.entomoljournal.com/archives/2018/vol6issue2/PartAJ/6-1-130-287.pdf .

[B9] Barrera-León M. Terán-Cabanillas E. Avena-Bustillos R. D. J. Cárdenas-Torres F. I. Amézquita-López B. A. Gómez-Favela M. A. (2025). Transformation of brewer’s spent grain through solid-state fermentation: implications for nutrition and health. Recycling 10, 170. 10.3390/recycling10050170

[B10] Bennett N. J. Cisneros-Montemayor A. M. Blythe J. Silver J. J. Singh G. Andrews N. (2019). Towards a sustainable and equitable blue economy. Nat. Sustain 2, 991–993. 10.1038/s41893-019-0404-1

[B11] Błaszczyk A. Augustyniak A. Skolimowski J. (2013). Ethoxyquin: an antioxidant used in animal feed. Int. J. Food Sci. 2013, 585931. 10.1155/2013/585931 26904606 PMC4745505

[B12] Bohnes F. A. Laurent A. (2019). LCA of aquaculture systems: methodological issues and potential improvements. Int. J. Life Cycle Assess. 24, 324–337. 10.1007/s11367-018-1517-x

[B13] Boissy J. Aubin J. Drissi A. van der Werf H. M. G. Bell G. J. Kaushik S. J. (2011). Environmental impacts of plant-based salmonid diets at feed and farm scales. Aquaculture 321, 61–70. 10.1016/j.aquaculture.2011.08.033

[B14] Boontiam W. Hong J. Kim Y.-Y. (2022). Dietary brewer grain meal with multienzymes supplementation affects growth performance, gut health, and antioxidative status of weaning pigs. Fermentation 8, 80. 10.3390/fermentation8020080

[B15] Boyd C. E. (2015). “Overview of aquaculture feeds: global impacts of ingredient use,” in Feed and feeding practices in aquaculture, (Elsevier), 3–25. Available online at: https://www.sciencedirect.com/science/article/pii/B9780081005064000015 (Accessed May 4, 2025).

[B16] Bravi E. Francesco G. D. Sileoni V. Perretti G. Galgano F. Marconi O. (2021). Brewing by-product upcycling potential: nutritionally valuable compounds and antioxidant activity evaluation. Antioxidants 10, 165. 10.3390/antiox10020165 33499399 PMC7911235

[B17] Cao L. Diana J. S. Keoleian G. A. (2013). Role of life cycle assessment in sustainable aquaculture. Rev. Aquac. 5, 61–71. 10.1111/j.1753-5131.2012.01080.x

[B18] Cárcamo D. Pérez T. Sánchez F. Oliver C. González L. Romero A. (2026). Brewer’s spent grain extract modulates the immune, intercellular junctions and antioxidant gene expression disrupting the Piscirickettsia salmonis infection in rainbow trout intestinal epithelial RTgutGC cells. Fish. Shellfish Immunol. 168, 110933. 10.1016/j.fsi.2025.110933 41076210

[B19] Carvalheiro F. Esteves M. P. Parajó J. C. Pereira H. Gırio F. M. (2004). Production of oligosaccharides by autohydrolysis of brewery’s spent grain. Bioresour. Technol. 91, 93–100. 10.1016/S0960-8524(03)00148-2 14585626

[B20] Casas-Godoy L. González-Escobar J. L. Mathis A. G. Barrera-Martínez I. (2023). Revalorization of untreated Brewer’s spent grain: novel and versatile feedstock to produce cellulases, lipases, and yeast biomass in a biorefinery approach. Biomass Convers. Biorefinery 13, 1659–1670. 10.1007/s13399-020-01157-3

[B21] Celus I. Brijs K. Delcour J. A. (2006). The effects of malting and mashing on barley protein extractability. J. Cereal Sci. 44, 203–211. 10.1016/j.jcs.2006.06.003

[B22] Chakraborty P. Mallik A. Sarang N. Lingam S. S. (2019). A review on alternative plant protein sources available for future sustainable aqua feed production. Int. J. Chem. Stud. 7, 1399–1404. Available online at: https://www.chemijournal.com/archives/2019/vol7issue3/PartW/7-2-252-301.pdf (Accessed May 5, 2025).

[B23] Chattaraj S. Behera B. K. Das Mohapatra P. K. (2024). Effective valorization of *Chlorella* biomass and brewers spent grain substituting fish meal and soybean meal in the diet of herbivorous fish *Cirrhinus reba* for higher growth, digestibility and sustainable cultivation. J. Appl. Phycol. 36, 217–232. 10.1007/s10811-023-03106-9

[B24] Chowdhury S. Saikia S. K. (2020). Oxidative stress in fish: a review. J. Sci. Res. 12, 145–160. 10.3329/jsr.v12i1.41716

[B25] Cian R. E. Hernández‐Chirlaque C. Gámez‐Belmonte R. Drago S. R. Sánchez De Medina F. Martínez‐Augustin O. (2020). Molecular action mechanism of anti‐inflammatory hydrolysates obtained from brewers’ spent grain. J. Sci. Food Agric. 100, 2880–2888. 10.1002/jsfa.10313 32020613

[B26] Ciji A. Akhtar M. S. (2021). Stress management in aquaculture: a review of dietary interventions. Rev. Aquac. 13, 2190–2247. 10.1111/raq.12565

[B27] Connolly A. Piggott C. O. FitzGerald R. J. (2013). Characterisation of protein-rich isolates and antioxidative phenolic extracts from pale and black brewers’ spent grain. Int. J. Food Sci. Technol. 48, 1670–1681. 10.1111/ijfs.12137

[B28] Costantini D. Metcalfe N. B. Monaghan P. (2010). Ecological processes in a hormetic framework. Ecol. Lett. 13, 1435–1447. 10.1111/j.1461-0248.2010.01531.x 20849442

[B29] Dabrowski K. Zhang Y. Kwasek K. Hliwa P. Ostaszewska T. (2010). Effects of protein-peptide- and free amino acid-based diets in fish nutrition. Aquac. Res. 41, 668–683. 10.1111/j.1365-2109.2010.02490.x

[B30] Dernekbasi S. Unal H. Karayucel I. Aral O. (2010). Effect of dietary supplementation of different rates of Spirulina (*Spirulina platensis*) on growth and feed conversion in guppy (*Poecilia reticulata* Peters, 1860). J. Anim. Vet. Adv. 9, 1395–1399. 10.3923/javaa.2010.1395.1399

[B31] Directorate General for Maritime Affairs and Fisheries and EUMOFA (2020). Recirculation aquaculture systems. Luxembourg: Publications office of the European Union. Available online at: https://data.europa.eu/doi/10.2771/66025 (Accessed May 9, 2025).

[B32] Division on Earth and Life Studies, Board on Agriculture and Natural Resources, Committee on the Nutrient Requirements of Fish and Shrimp (2021). ““Proteins and Amino Acids,”,” in Nutrient requirements of fish and shrimp (Washington, D.C.: National Academies Press), 57–101.

[B33] Eche V. Emenike C. U. Rupasinghe H. P. V. Eche V. Emenike C. U. Rupasinghe H. P. V. (2025). Nutritional value of Brewer’s spent grain and consumer acceptance of its value-added food products. Foods 14, 2900. 10.3390/foods14162900 40870812 PMC12386034

[B34] El-Sayed A.-F. M. (2013). “On-farm feed management practices for nile tilapia (*Oreochromis niloticus*) in Egypt,” in On-farm feeding and feed management in aquaculture. Editors Hasan M. R. New M. B. (Rome: FAO), 101–129.

[B35] Estevão-Rodrigues T. Fernandes H. Moutinho S. Filipe D. Fontinha F. Magalhães R. (2024). Effect of solid-state fermentation of Brewer’s spent grain on digestibility and digestive function of European seabass (*Dicentrarchus labrax*) juveniles. Anim. Feed Sci. Technol. 315, 116018. 10.1016/j.anifeedsci.2024.116018

[B36] Estevão-Rodrigues T. Fernandes H. Moutinho S. Couto A. Belo I. Castro C. (2025a). Fermented brewer’s spent grain as a sustainable feed ingredient impacts meagre (*Argyrosomus regius*) growth, intermediary metabolism, digestive enzymes, oxidative stress, and intestinal histology. Animal Feed Sci. Technol. 326, 116388. 10.1016/j.anifeedsci.2025.116388

[B37] Estevão-Rodrigues T. Fernandes H. Moutinho S. Ferreira M. Castro C. Belo I. (2025b). Effect of solid-fermented brewer’s spent grain on growth, metabolism, and oxidative status of European seabass (*Dicentrarchus labrax*). Fishes 10, 49. 10.3390/fishes10020049

[B38] Estévez A. Padrell L. Iñarra B. Orive M. San Martin D. (2021). Brewery by-products (yeast and spent grain) as protein sources in gilthead seabream (*Sparus aurata*) feeds. Aquaculture 543, 736921. 10.1016/j.aquaculture.2021.736921

[B39] Estévez A. Padrell L. Iñarra B. Orive M. San Martin D. (2022). Brewery by-products (yeast and spent grain) as protein sources in rainbow trout (*Oncorhynchus mykiss*) feeds. Front. Mar. Sci. 9, 862020. 10.3389/fmars.2022.862020

[B40] Eurostat (2024). 34.3 bn litres of beer produced in the EU in 2023. Available online at: https://ec.europa.eu/eurostat/web/products-eurostat-news/w/edn-20240802-1 (Accessed May 5, 2025).

[B41] FAO (2024). The state of world fisheries and aquaculture 2024 - blue transformation in action. Rome: FAO. 10.4060/cd0683en

[B42] FAO (2025). FAO: nile tilapia home. Available online at: https://www.fao.org/fishery/affris/species-profiles/nile-tilapia/nile-tilapia-home/en/ (Accessed May 7, 2025).

[B43] Fernandes H. Moyano F. Castro C. Salgado J. Martínez F. Aznar M. (2021). Solid-state fermented brewer’s spent grain enzymatic extract increases *in vitro* and *in vivo* feed digestibility in European seabass. Sci. Rep. 11, 22946. 10.1038/s41598-021-02393-x 34824341 PMC8617204

[B44] Fernandes H. Castro C. Filipe D. Ferreira P. Salgado J. M. Moyano F. (2022). Application of fermented Brewer’s spent grain extract in plant-based diets improves pre- and post-mortem oxidative status of European seabass (*Dicentrarchus labrax*). Aquac. Nutr. 2022, 2629052. 10.1155/2022/2629052

[B45] Freitas L. C. Barbosa J. R. da Costa A. L. C. Bezerra F. W. F. Pinto R. H. H. de Carvalho Junior R. N. (2021). From waste to sustainable industry: how can agro-industrial wastes help in the development of new products? Resour. Conserv. Recycl. 169, 105466. 10.1016/j.resconrec.2021.105466

[B46] Fréon P. Durand H. Avadí A. Huaranca S. Orozco Moreyra R. (2017). Life cycle assessment of three Peruvian fishmeal plants: toward a cleaner production. J. Clean. Prod. 145, 50–63. 10.1016/j.jclepro.2017.01.036

[B47] Gandolpho B. C. G. Almeida A. da R. Gandolpho G. de M. Gasparini O. C. Ferreira B. L. Zanetti M. B. R. (2025). Trub, a brewing byproduct, is an innovative and valuable source of nutrients and natural antioxidants viable for fish dietary supplementation. J. Am. Soc. Brew. Chem. 83, 192–202. 10.1080/03610470.2024.2411652

[B48] Gatlin III D. M. Barrows F. T. Brown P. Dabrowski K. Gaylord T. G. Hardy R. W. (2007). Expanding the utilization of sustainable plant products in aquafeeds: a review. Aquac. Res. 38, 551–579. 10.1111/j.1365-2109.2007.01704.x

[B49] Geremia E. Ripa M. Catone C. M. Ulgiati S. (2021). A review about microalgae wastewater treatment for bioremediation and biomass production—A new challenge for Europe. Environments 8, 136. 10.3390/environments8120136

[B50] Ghamkhar R. Hicks A. (2020). Comparative environmental impact assessment of aquafeed production: sustainability implications of forage fish meal and oil free diets. Resour. Conserv. Recycl. 161, 104849. 10.1016/j.resconrec.2020.104849

[B51] Glencross B. D. Bachis E. Robb D. Newton R. (2024). TThe evolution of sustainability metrics for the marine ingredient sector: moving towards holistic assessments of aquaculture feed. Rev. Fish. Sci. Aquac. 0, 1–17. 10.1080/23308249.2024.2337426

[B52] Glowka R. P. Weingartner M. Boscolo W. R. (2025). Determination of apparent digestibility coefficients of brewery waste for nile tilapia. OBS. De. LA Econ. LATINOAM. 23, e8951. 10.55905/oelv23n2-046

[B53] Gutiérrez-Barrutia M. B. del Castillo M. D. Arcia P. Cozzano S. (2022). Brewer’s spent grain as a food ingredient for a healthy, safe, and sustainable human diet. Foods 11, 1403. 10.3390/foods11101403 35626975 PMC9140782

[B54] Han P. Lu Q. Fan L. Zhou W. (2019). A review on the use of microalgae for sustainable aquaculture. Appl. Sci. 9, 2377. 10.3390/app9112377

[B55] Hardy R. W. Kaushik S. J. (2021). Fish nutrition. Academic Press. Available online at: https://books.google.com/books?hl=it&lr=&id=CF8BEAAAQBAJ&oi=fnd&pg=PP1&dq=Hardy,+R.W.%3B+Kaushik,+S.J.+Fish+Nutrition&ots=GOpjZNhmQs&sig=a69rzpqhIXZrbIL2tjXQEbsFRHg (Accessed May 6, 2025).

[B56] Hartman G. L. West E. D. Herman T. K. (2011). Crops that feed the world 2. Soybean—worldwide production, use, and constraints caused by pathogens and pests. Food secur. 3, 5–17. 10.1007/s12571-010-0108-x

[B57] Hassan M. A. Aftabuddin Md. Meena D. K. Mishal P. Gupta S. D. (2016). Effective utilization of distiller’s grain soluble—an agro-industrial waste in the feed of cage-reared minor carp *Labeo bata* in a tropical reservoir, India. Environ. Sci. Pollut. Res. 23, 16090–16095. 10.1007/s11356-016-6732-z 27146546

[B58] He Y. Kuhn D. D. Ogejo J. A. O’Keefe S. F. Fraguas C. F. Wiersema B. D. (2019). Wet fractionation process to produce high protein and high fiber products from brewer’s spent grain. Food Bioprod. process. 117, 266–274. 10.1016/j.fbp.2019.07.011

[B59] He Y. Galagarza O. A. Wang H. Taylor Z. W. Ferguson C. S. Ogejo J. A. (2020). Protein-rich product recovered from brewer’s spent grain can partially replace fishmeal in diets of Pacific white shrimp, *Litopenaeus vannamei* . Aquac. Res. 51, 3284–3296. 10.1111/are.14664

[B60] He Y. Allen J. Huang H. (2023). “4.25 - food by-products valorization technologies: brewer’s spent grain,” in Sustainable food science - a comprehensive approach. Editor Ferranti P. (Oxford: Elsevier), 447–463. 10.1016/B978-0-12-823960-5.00091-3

[B61] Hospido A. Tyedmers P. (2005). Life cycle environmental impacts of Spanish tuna fisheries. Fish. Res. 76, 174–186. 10.1016/j.fishres.2005.05.016

[B62] Hu X. Ma W. Zhang D. Tian Z. Yang Y. Huang Y. (2025). Application of natural antioxidants as feed additives in aquaculture: a review. Biology 14, 87. 10.3390/biology14010087 39857317 PMC11762552

[B63] IAFFD (2025). IAFFD (The international aquaculture feed formulation database). Available online at: https://iaffd.com (Accessed May 6, 2025).

[B64] IFFO (2025). FIFO data. Available online at: https://www.iffo.com/fifo-data (Accessed May 7, 2025).

[B65] Ikram S. Huang L. Zhang H. Wang J. Yin M. (2017). Composition and nutrient value proposition of brewers spent grain. J. Food Sci. 82, 2232–2242. 10.1111/1750-3841.13794 28833108

[B66] ISO (2006). Environmental management–life cycle assessment–principles and framework. Geneva: ISO.

[B67] Jay A. J. Parker M. L. Faulks R. Husband F. Wilde P. Smith A. C. (2008). A systematic micro-dissection of brewers’ spent grain. J. Cereal Sci. 47, 357–364. 10.1016/j.jcs.2007.05.006

[B68] Jayant M. Hassan M. A. Srivastava P. P. Meena D. K. Kumar P. Kumar A. (2018). Brewer’s spent grains (BSGs) as feedstuff for striped catfish, *Pangasianodon hypophthalmus* fingerlings: an approach to transform waste into wealth. J. Clean. Prod. 199, 716–722. 10.1016/j.jclepro.2018.07.213

[B69] Jeney G. (2017). Fish diseases: prevention and control strategies. Academic Press. Available online at: https://www.perlego.com/book/1829928/fish-diseases-prevention-and-control-strategies-pdf (Accessed May 6, 2025).

[B70] Jia R. Wang L. Hou Y. Feng W. Li B. Zhu J. (2022). Effects of stocking density on the growth performance, physiological parameters, redox status and lipid metabolism of *Micropterus salmoides* in integrated rice–fish farming systems. Antioxidants 11, 1215. 10.3390/antiox11071215 35883706 PMC9312047

[B71] Jiang Q. Bhattarai N. Pahlow M. Xu Z. (2022). Environmental sustainability and footprints of global aquaculture. Resour. Conserv. Recycl. 180, 106183. 10.1016/j.resconrec.2022.106183

[B72] Jin Z. Lan Y. Ohm J.-B. Gillespie J. Schwarz P. Chen B. (2022). Physicochemical composition, fermentable sugars, free amino acids, phenolics, and minerals in brewers’ spent grains obtained from craft brewing operations. J. Cereal Sci. 104, 103413. 10.1016/j.jcs.2022.103413

[B73] Johnson P. Paliwal J. Cenkowski S. (2010). Issues with utilisation of brewers’ spent grain. Stewart Postharvest Rev. 6, 1–8. 10.2212/spr.2010.4.2

[B74] Kanauchi O. Mitsuyama K. Araki Y. (2001). Development of a functional germinated barley foodstuff from brewer’s spent grain for the treatment of ulcerative colitis. J. Am. Soc. Brew. Chem. 59, 59–62. 10.1094/ASBCJ-59-0059

[B75] Kandeel M. M. A. Magouz F. I. Omar A. A. Amer A. A. Zaineldin A. I. Ashry A. M. (2024). Combined effects of butyl hydroxytoluene and vitamin C on the growth performance, blood biochemistry, and antioxidative status of common carp (*Cyprinus carpio*). Ann. Anim. Sci. 24, 881–888. 10.2478/aoas-2024-0014

[B76] Karapanagiotidis I. T. Metsoviti M. N. Gkalogianni E. Z. Psofakis P. Asimaki A. Katsoulas N. (2022). The effects of replacing fishmeal by *Chlorella vulgaris* and fish oil by *Schizochytrium* sp. and *Microchloropsis gaditana* blend on growth performance, feed efficiency, muscle fatty acid composition and liver histology of gilthead seabream (*Sparus aurata*). Aquaculture 561, 738709. 10.1016/j.aquaculture.2022.738709

[B77] Karlsen F. Skov P. V. (2022). Review – potentials and limitations of utilising brewer’s spent grain as a protein source in aquaculture feeds. J. Clean. Prod. 357, 131986. 10.1016/j.jclepro.2022.131986

[B78] Kaur V. I. Saxena P. K. (2004). Incorporation of brewery waste in supplementary feed and its impact on growth in some carps. Bioresour. Technol. 91, 101–104. 10.1016/S0960-8524(03)00073-7 14585627

[B79] Kok B. Malcorps W. Tlusty M. F. Eltholth M. M. Auchterlonie N. A. Little D. C. (2020). Fish as feed: using economic allocation to quantify the fish in: fish out ratio of major fed aquaculture species. Aquaculture 528, 735474. 10.1016/j.aquaculture.2020.735474

[B80] Kokou F. Fountoulaki E. (2018). Aquaculture waste production associated with antinutrient presence in common fish feed plant ingredients. Aquaculture 495, 295–310. 10.1016/j.aquaculture.2018.06.003

[B81] Kononova S. V. Zinchenko D. V. Muranova T. A. Belova N. A. Miroshnikov A. I. (2019). Intestinal microbiota of salmonids and its changes upon introduction of soy proteins to fish feed. Aquac. Int. 27, 475–496. 10.1007/s10499-019-00341-1

[B82] Krogdahl Å. Penn M. Thorsen J. Refstie S. Bakke A. M. (2010). Important antinutrients in plant feedstuffs for aquaculture: an update on recent findings regarding responses in salmonids. Aquac. Res. 41, 333–344. 10.1111/j.1365-2109.2009.02426.x

[B83] Kumari B. Tiwari B. K. Walsh D. Griffin T. P. Islam N. Lyng J. G. (2019). Impact of pulsed electric field pre-treatment on nutritional and polyphenolic contents and bioactivities of light and dark brewer’s spent grains. Innov. Food Sci. Emerg. Technol. 54, 200–210. 10.1016/j.ifset.2019.04.012

[B84] Lu Q. Li H. Zou Y. Liu H. Yang L. (2021). Astaxanthin as a microalgal metabolite for aquaculture: a review on the synthetic mechanisms, production techniques, and practical application. Algal Res. 54, 102178. 10.1016/j.algal.2020.102178

[B85] Lundebye A.-K. Hove H. Måge A. Bohne V. J. B. Hamre K. (2010). Levels of synthetic antioxidants (ethoxyquin, butylated hydroxytoluene and butylated hydroxyanisole) in fish feed and commercially farmed fish. Food Addit. Contam. Part A 27, 1652–1657. 10.1080/19440049.2010.508195 20931417

[B86] Lushchak V. I. (2011). Environmentally induced oxidative stress in aquatic animals. Aquat. Toxicol. 101, 13–30. 10.1016/j.aquatox.2010.10.006 21074869

[B87] Lushchak V. I. (2016). Contaminant-induced oxidative stress in fish: a mechanistic approach. Fish. Physiol. Biochem. 42, 711–747. 10.1007/s10695-015-0171-5 26607273

[B88] Lynch F. Santana-Sánchez A. Jämsä M. Sivonen K. Aro E.-M. Allahverdiyeva Y. (2015). Screening native isolates of Cyanobacteria and a green alga for integrated wastewater treatment, biomass accumulation and neutral lipid production. Algal Res. 11, 411–420. 10.1016/j.algal.2015.05.015

[B89] MacLeod M. Hasan M. R. Robb D. H. Mamun-Ur-Rashid M. (2019). Quantifying and mitigating greenhouse gas emissions from global aquaculture. Rome: Food and Agriculture Organization of the United Nations.

[B90] Mantovani M. Marazzi F. Fornaroli R. Bellucci M. Ficara E. Mezzanotte V. (2020). Outdoor pilot-scale raceway as a microalgae-bacteria sidestream treatment in a WWTP. Sci. Total Environ. 710, 135583. 10.1016/j.scitotenv.2019.135583 31785903

[B91] Martínez-Antequera F. P. Simó-Mirabet P. Heras V. Román M. Mancera J. M. Martos-Sitcha J. A. (2025). Effect of two different pretreatments of brewers spent grain used as feed ingredient on nutritional, immunological, and metabolical parameters in gilthead seabream (*Sparus aurata*). Biology 14, 585. 10.3390/biology14060585 40563837 PMC12189433

[B92] McCarthy A. L. O’Callaghan Y. C. Connolly A. Piggott C. O. FitzGerald R. J. O’Brien N. M. (2013). *In vitro* antioxidant and anti-inflammatory effects of brewers’ spent grain protein rich isolate and its associated hydrolysates. Food Res. Int. 50, 205–212. 10.1016/j.foodres.2012.10.022

[B93] McCarthy A. L. O’Callaghan Y. C. Connolly A. Piggott C. O. FitzGerald R. J. O’Brien N. M. (2014). Phenolic-enriched fractions from brewers’ spent grain possess cellular antioxidant and immunomodulatory effects in cell culture model systems. J. Sci. Food Agric. 94, 1373–1379. 10.1002/jsfa.6421 24114648

[B94] Mekonnen M. M. Gerbens-Leenes W. (2020). The water footprint of global food production. Water 12, 2696. 10.3390/w12102696

[B95] Mennaa F. Z. Arbib Z. Perales J. A. (2015). Urban wastewater treatment by seven species of microalgae and an algal bloom: biomass production, N and P removal kinetics and harvestability. Water Res. 83, 42–51. 10.1016/j.watres.2015.06.007 26117372

[B96] Merten D. Erman L. Marabelli G. P. Leners B. Ney Y. Nasim M. J. (2022). Potential health effects of brewers’ spent grain as a functional food ingredient assessed by markers of oxidative stress and inflammation following gastro-intestinal digestion and in a cell model of the small intestine. Food Funct. 13, 5327–5342. 10.1039/d1fo03090f 35446320

[B97] Mitra A. (2021). Thought of alternate aquafeed: conundrum in aquaculture sustainability? Proc. Zool. Soc. 74, 1–18. 10.1007/s12595-020-00352-4

[B98] Mussatto S. I. Roberto I. C. (2005). Acid hydrolysis and fermentation of brewer’s spent grain to produce xylitol. J. Sci. Food Agric. 85, 2453–2460. 10.1002/jsfa.2276

[B99] Mussatto S. I. Dragone G. Roberto I. C. (2006). Brewers’ spent grain: generation, characteristics and potential applications. J. Cereal Sci. 43, 1–14. 10.1016/j.jcs.2005.06.001

[B100] Napolitano G. Fasciolo G. Venditti P. (2021). Mitochondrial management of reactive oxygen species. Antioxidants 10, 1824. 10.3390/antiox10111824 34829696 PMC8614740

[B101] Napolitano G. Fasciolo G. Venditti P. (2022a). The ambiguous aspects of oxygen. Oxygen 2, 382–409. 10.3390/oxygen2030027

[B102] Napolitano G. Venditti P. Agnisola C. Quartucci S. Fasciolo G. Muscari Tomajoli M. T. (2022b). Towards sustainable aquaculture systems: biological and environmental impact of replacing fishmeal with *Arthrospira platensis* (Nordstedt) (spirulina). J. Clean. Prod. 374, 133978. 10.1016/j.jclepro.2022.133978

[B103] Napolitano G. Capriello T. Venditti P. Fasciolo G. La Pietra A. Trifuoggi M. (2023). Aluminum induces a stress response in zebrafish gills by influencing metabolic parameters, morphology, and redox homeostasis. Comp. Biochem. Physiol. C. Toxicol. Pharmacol. 271, 109633. 10.1016/j.cbpc.2023.109633 37084860

[B104] Nawaz A. Bakhsh javaid A. Irshad S. Hoseinifar S. H. Xiong H. (2018). The functionality of prebiotics as immunostimulant: evidences from trials on terrestrial and aquatic animals. Fish. Shellfish Immunol. 76, 272–278. 10.1016/j.fsi.2018.03.004 29510254

[B105] Nazzaro J. San Martin D. Perez-Vendrell A. M. Padrell L. Iñarra B. Orive M. (2021). Apparent digestibility coefficients of brewer’s by-products used in feeds for rainbow trout (*Oncorhynchus mykiss*) and gilthead seabream (*Sparus aurata*). Aquaculture 530, 735796. 10.1016/j.aquaculture.2020.735796

[B106] Nganyira P. D. Mahushi D. J. Balengayabo J. G. Shao G. N. Emmanuel J. K. (2023). Quality of biogas generated through co-digestion of Brewer’s spent grain and cattle dung. Energy Rep. 10, 2330–2336. 10.1016/j.egyr.2023.09.012

[B107] Nganyira P. D. Mahushi D. J. Balengayabo J. G. Shao G. N. Emmanuel J. K. (2025). Quantity of biogas produced through co-digestion of Brewer’s spent grain and cattle dung. Biofuels 16 (10), 1116–1122. 10.1080/17597269.2025.2487734

[B108] Nimalan N. Sørensen S. L. Fečkaninová A. Koščová J. Mudroňová D. Gancarčíková S. (2022). Mucosal barrier status in Atlantic salmon fed marine or plant-based diets supplemented with probiotics. Aquaculture 547, 737516. 10.1016/j.aquaculture.2021.737516

[B109] Noga E. J. (2010). Fish disease: diagnosis and treatment. John Wiley and Sons. Available online at: https://books.google.com/books?hl=it&lr=&id=K5-HDwAAQBAJ&oi=fnd&pg=PR9&dq=Fish+Disease%E2%80%AF:+Diagnosis+and+Treatment&ots=l-1PTBcZxb&sig=xu3etLM-orHBTid7mAqSHCth00I (Accessed May 6, 2025).

[B110] Obirikorang K. A. Amisah S. Fialor S. C. Skov P. V. (2015). Local agro-industrial by-products with potential use in Ghanaian aquaculture: a review. Aquac. Int. 23, 403–425. 10.1007/s10499-014-9831-1

[B111] Oliva M. E. Cian R. E. Ferreira M. D. R. Garzón A. G. Drago S. R. D’Alessandro M. E. (2023). *In vivo* and *in silico* study of antioxidant and anti-inflammatory effects on the liver-spleen axis of microencapsulated brewers’ spent grain peptides. Food Funct. 14, 5290–5300. 10.1039/D2FO04104A 37195630

[B112] Oliva-Teles A. Gonçalves P. (2001). Partial replacement of fishmeal by brewers yeast (*Saccaromyces cerevisae*) in diets for sea bass (*Dicentrarchus labrax*) juveniles. Aquaculture 202, 269–278. 10.1016/S0044-8486(01)00777-3

[B113] Olsen M. L. Olsen K. Jensen P. E. (2024). Consumer acceptance of microalgae as a novel food - where are we now? And how to get further. Physiol. Plant. 176, e14337. 10.1111/ppl.14337 38716544

[B114] Orzel D. Mazurek D. Laszczak E. Figurska Ciura D. Lozna K. Styczynska M. (2018). Influence of brewer’s spent grain added to high fat diet with lard on selected biochemical blood indicators in Wistar rats. J. Food Process. Technol. 9, 732. 10.4172/2157-7110.1000732

[B115] Osório J. Stiller K. T. Reiten B.-K. Kolarevic J. Johansen L.-H. Afonso F. (2022). Intermittent administration of peracetic acid is a mild environmental stressor that elicits mucosal and systemic adaptive responses from Atlantic salmon post-smolts. BMC Zool. 7, 1. 10.1186/s40850-021-00100-x 37170301 PMC10127346

[B116] Patel A. K. Singhania R. R. Awasthi M. K. Varjani S. Bhatia S. K. Tsai M.-L. (2021). Emerging prospects of macro- and microalgae as prebiotic. Microb. Cell Fact. 20, 112. 10.1186/s12934-021-01601-7 34090444 PMC8180151

[B117] Pechsiri J. S. Thomas J.-B. E. Bahraoui N. E. Fernandez F. G. A. Chaouki J. Chidami S. (2023). Comparative life cycle assessment of conventional and novel microalgae production systems and environmental impact mitigation in urban-industrial symbiosis. Sci. Tot. Environ. 854, 158445. 10.1016/j.scitotenv.2022.158445 36058335

[B118] Pelletier N. Tyedmers P. (2007). Feeding farmed salmon: is organic better? Aquaculture 272, 399–416. 10.1016/j.aquaculture.2007.06.024

[B119] Pereira R. Costa M. Velasco C. Cunha L. M. Lima R. C. Baião L. F. (2022). Comparative analysis between synthetic vitamin E and natural antioxidant sources from tomato, carrot and coriander in diets for market-sized *Dicentrarchus labrax* . Antioxidants 11, 636. 10.3390/antiox11040636 35453321 PMC9030101

[B120] Peres H. Oliva-Teles A. (2005). The effect of dietary protein replacement by crystalline amino acid on growth and nitrogen utilization of turbot *Scophthalmus maximus* juveniles. Aquaculture 250, 755–764. 10.1016/j.aquaculture.2005.04.046

[B121] Petereit J. Hoerterer C. Bischoff-Lang A. A. Conceição L. E. C. Pereira G. Johansen J. (2022). Adult European seabass (*Dicentrarchus labrax*) perform well on alternative circular-economy-driven feed formulations. Sustainability 14, 7279. 10.3390/su14127279

[B122] Prescott S. G. (2017). Exploring the sustainability of open-water marine, integrated multi-trophic aquaculture, using life-cycle assessment. University of Stirling. Available online at: https://api.semanticscholar.org/CorpusID:135383352 (Accessed May 4, 2025).

[B123] Ragaza J. A. Hossain Md. S. Meiler K. A. Velasquez S. F. Kumar V. (2020). A review on Spirulina: alternative media for cultivation and nutritive value as an aquafeed. Rev. Aquac. 12, 2371–2395. 10.1111/raq.12439

[B124] Robertson J. A. I’Anson K. J. A. Treimo J. Faulds C. B. Brocklehurst T. F. Eijsink V. G. H. (2010). Profiling brewers’ spent grain for composition and microbial ecology at the site of production. LWT - Food Sci. Technol. 43, 890–896. 10.1016/j.lwt.2010.01.019

[B125] Rojas-Chamorro J. A. Romero I. López-Linares J. C. Castro E. (2020). Brewer’s spent grain as a source of renewable fuel through optimized dilute acid pretreatment. Renew. Energy 148, 81–90. 10.1016/j.renene.2019.12.030

[B126] Rollin X. Mambrini M. Abboudi T. Larondelle Y. Kaushik S. J. (2003). The optimum dietary indispensable amino acid pattern for growing Atlantic salmon (*Salmo salar* L.) fry. Br. J. Nutr. 90, 865–876. 10.1079/BJN2003973 14667180

[B127] Roy S. S. Pal R. (2015). Microalgae in aquaculture: a review with special references to nutritional value and fish dietetics. Proc. Zool. Soc. 68, 1–8. 10.1007/s12595-013-0089-9

[B128] San Martin D. Orive M. Iñarra B. Castelo J. Estévez A. Nazzaro J. (2020). Brewers’ spent yeast and grain protein hydrolysates as second-generation feedstuff for aquaculture feed. Waste Biomass Valorization 11, 5307–5320. 10.1007/s12649-020-01145-8

[B129] Sarker P. K. Kapuscinski A. R. McKuin B. Fitzgerald D. S. Nash H. M. Greenwood C. (2020). Microalgae-blend tilapia feed eliminates fishmeal and fish oil, improves growth, and is cost viable. Sci. Rep. 10, 19328. 10.1038/s41598-020-75289-x 33184333 PMC7665073

[B130] Schettino R. Verni M. Acin-Albiac M. Vincentini O. Krona A. Knaapila A. (2021). Bioprocessed brewers’ spent grain improves nutritional and antioxidant properties of pasta. Antioxidants 10, 742. 10.3390/antiox10050742 34067199 PMC8151577

[B131] Shi Y. Zhong L. Fan Y. Zhang J. Zhong H. Liu X. (2022). The protective effect of mulberry leaf flavonoids on high-carbohydrate-induced liver oxidative stress, inflammatory response and intestinal microbiota disturbance in *Monopterus albus* . Antioxidants 11, 976. 10.3390/antiox11050976 35624840 PMC9137898

[B132] Singh R. Das R. Sangwan S. Rohatgi B. Khanam R. Peera S. K. P. G. (2021). Utilisation of agro-industrial waste for sustainable green production: a review. Environ. Sustain. 4, 619–636. 10.1007/s42398-021-00200-x

[B133] Song C. Sun C. Liu B. Xu P. (2023). Oxidative stress in aquatic organisms. Antioxidants 12, 1223. 10.3390/antiox12061223 37371953 PMC10295492

[B134] Statista (2025). Worldwide beer production 2023. Statista. Available online at: https://www.statista.com/statistics/270275/worldwide-beer-production/ (Accessed May 5, 2025).

[B135] Stickney R. R. Gatlin I. I. I. D. (2022). Aquaculture. An introductory text. 4th Edition. Boston, MA: CABI. Available online at: https://www.cabidigitallibrary.org/doi/book/10.1079/9781800621145.0000 (Accessed May 4, 2025).

[B136] Subaramaniyam U. Allimuthu R. S. Vappu S. Ramalingam D. Balan R. Paital B. (2023). Effects of microplastics, pesticides and nano-materials on fish health, oxidative stress and antioxidant defense mechanism. Front. Physiol. 14, 1217666. 10.3389/fphys.2023.1217666 37435307 PMC10331820

[B137] Subba R. Fasciolo G. Geremia E. Muscari Tomajoli M. T. Petito A. Carrella S. (2024). Simultaneous induction of systemic hyperglycaemia and stress impairs brain redox homeostasis in the adult zebrafish. Arch. Biochem. Biophys. 759, 110101. 10.1016/j.abb.2024.110101 39029645

[B138] Thrane M. (2006). LCA of Danish fish products. New methods and insights. Int. J. Life Cycle Assess. 11, 66–74. 10.1065/lca2006.01.232

[B139] Tidwell J. H. Coyle S. D. Rossi W. Rucker K. (2023). Evaluation of brewers spent grains with different levels of exogenous enzymes on the production performance and body composition of Nile tilapia (*Oreochromis niloticus*) and channel catfish (*Ictalurus punctatus*). J. Appl. Aquac. 35, 257–272. 10.1080/10454438.2021.1956669

[B140] Tsiplakou E. Pitino R. Manuelian C. L. Simoni M. Mitsiopoulou C. De Marchi M. (2021). Plant feed additives as natural alternatives to the use of synthetic antioxidant vitamins in livestock animal products yield, quality, and oxidative status: a review. Antioxidants 10, 780. 10.3390/antiox10050780 34069000 PMC8155892

[B141] Tzachor A. Smidt-Jensen A. Ramel A. Geirsdóttir M. (2022). Environmental impacts of large-scale spirulina (*Arthrospira platensis*) production in hellisheidi geothermal park Iceland: life cycle assessment. Mar. Biotechnol. 24, 991–1001. 10.1007/s10126-022-10162-8 36071348 PMC9560931

[B142] Vázquez-Romero B. Villar-Navarro E. Perales J. A. Garrido-Pérez C. Ruiz J. (2024). Techno-economic analysis of using microalgae to treat streams from fish RAS farming and replace fish meal: a case study. J. Water Process Eng. 59, 104904. 10.1016/j.jwpe.2024.104904

[B143] Walsh P. P. Banerjee A. Murphy E. (2022). “The UN 2030 agenda for sustainable development,” in Partnerships and the sustainable development goals. Editors Murphy E. Banerjee A. Walsh P. P. (Cham: Springer International Publishing), 1–12. 10.1007/978-3-031-07461-5_1

[B144] Waters D. M. Jacob F. Titze J. Arendt E. K. Zannini E. (2012). Fibre, protein and mineral fortification of wheat bread through milled and fermented brewer’s spent grain enrichment. Eur. Food Res. Technol. 235, 767–778. 10.1007/s00217-012-1805-9

[B145] Webster C. D. Lim C. (2002). Nutrient requirements and feeding of finfish for aquaculture (Boston, MA: CABI).

[B146] Wen C. Zhang J. Duan Y. Zhang H. Ma H. (2019). A mini-review on brewer’s spent grain protein: isolation, physicochemical properties, application of protein, and functional properties of hydrolysates. J. Food Sci. 84, 3330–3340. 10.1111/1750-3841.14906 31834967

[B147] Williams K. Barlow C. Rodgers L. (2001). Efficacy of crystalline and protein-bound amino acids for amino acid enrichment of diets for barramundi/Asian seabass (*Lates calcarifer* Bloch). Aquac. Res. 32, 415–429. 10.1046/j.1355-557x.2001.00032.x

[B148] Wuang S. C. Khin M. C. Chua P. Q. D. Luo Y. D. (2016). Use of *Spirulina* biomass produced from treatment of aquaculture wastewater as agricultural fertilizers. Algal Res. 15, 59–64. 10.1016/j.algal.2016.02.009

[B149] Xing S. Liang X. Zhang X. Oliva-Teles A. Peres H. Li M. (2024). Essential amino acid requirements of fish and crustaceans, a meta-analysis. Rev. Aquac. 16, 1069–1086. 10.1111/raq.12886

[B150] Xiros C. Christakopoulos P. (2012). Biotechnological potential of brewers spent grain and its recent applications. Waste Biomass Valor 3, 213–232. 10.1007/s12649-012-9108-8

[B151] Zeko-Pivač A. Tišma M. Žnidaršič-Plazl P. Kulisic B. Sakellaris G. Hao J. (2022). The potential of brewer’s spent grain in the circular bioeconomy: state of the art and future perspectives. Front. Bioeng. Biotechnol. 10, 870744. 10.3389/fbioe.2022.870744 35782493 PMC9247607

[B152] Zerai D. B. Fitzsimmons K. M. Collier R. J. Duff G. C. (2008). Evaluation of brewer’s waste as partial replacement of fish meal protein in Nile tilapia, *Oreochromis niloticus*, diets. J. World Aquac. Soc. 39, 556–564. 10.1111/j.1749-7345.2008.00186.x

[B153] Zhang J. Sundfør E. B. Klokkerengen R. Gonzalez S. V. Mota V. C. Lazado C. C. (2022). Determination of the oxidative stress biomarkers of 8-hydroxydeoxyguanosine and dityrosine in the gills, skin, dorsal fin, and liver tissue of Atlantic salmon (*Salmo salar*) Parr. Toxics 10, 509. 10.3390/toxics10090509 36136474 PMC9503732

[B154] Ziegler F. Nilsson P. Mattsson B. Walther Y. (2003). Life cycle assessment of frozen cod fillets including fishery-specific environmental impacts. Int. J. Life Cycle Assess. 8, 39–47. 10.1007/BF02978747

