# Supplementary material for: Potential opportunities and challenges of utilizing brewer’s spent grain in sustainable fish feeding within the circular economy
Source: Front Physiol. 2026 Mar 18;17:1766656. doi: 10.3389/fphys.2026.1766656 (PMC13038440; doi:10.3389/fphys.2026.1766656)
Supplement: Supplementary file 2 [file DataSheet1.docx]

Potential Opportunities and Challenges of Utilizing Brewer’s Spent Grain in Sustainable Fish Feeding within the Circular Economy

Supplementary Material 1

# Supplementary Data

## Analysis of the potential environmental impacts of using BSG for fish feeding in aquaculture

### Aims of the analysis

In this analysis, we investigated whether replacing fishmeal, wheat flour, or soybean meal with BSG can reduce environmental impacts. As a reference diet, we used the one reported for Nile Tilapia (*Oreochromis niloticus* L.) by Zerai et al. (2008) (Control diet, Supplementary Table 1). We determined the necessary reductions in the other ingredients required to maintain the balance of nutrients when adding 10% and 37% BSG to the diet (diets D1 and D2, respectively, Supplementary Table 1). Formulation was made using AFOS (Animal Feed Formulation Software, [www.animalfeedsoftware.com)](http://www.animalfeedsoftware.com/) and the composition data from the IAFFD database (IAFFD, no date). Formulation and composition are reported in Tables 1 and 2, respectively. We applied the Life Cycle Assessment (LCA; ISO, 2006) analysis to them to evaluate the global and detailed environmental impacts associated with their production. We also calculated the FIFO values of the three diets.

### Life Cycle Assessment

LCA is a standardized tool that evaluates the environmental impacts of a product or service by considering all phases of its life cycle, from the extraction of raw materials to production, use, and maintenance, as well as reuse and waste disposal. Environmental impacts can arise from the resources extracted from the environment (input) and the emissions released (output) during each phase of the process (Huijbregts et al., 2016).

LCA was applied to the three diets described in Supplementary Table 1 using the software Simapro (PRé Sustainability B.V., Amersfoort, The Netherlands) and two different databases: Ecoinvent and Agrifootprint, as well as Ecoinvent 3. The impact analysis used the ReCiPe Midpoint (H) method (Huijbregts et al., 2016). This method transforms the life cycle inventory results into a set of environmental impact indicators. The ReCiPe method includes two classes of indicators: 18 midpoint indicators and three endpoint indicators. We decided to use the midpoint indicators class, which can offer precise and detailed results per unit of measurement.

This approach aimed to identify, quantify, and evaluate the environmental impacts associated with the production of the three different diets. One kilogram of feed was chosen as the Functional Unit. The feed inventory considers all ingredients required to make the diets, excluding electricity consumption, which is primarily due to pellet production and is equal across all three diets. It would dominate the analysis, hiding all other impacts. BSG is not available as a dedicated process in the Ecoinvent database. Therefore, we modeled BSG using the most similar process available, namely DDGS (Distiller's Dried Grains with Solubles), derived from ethanol production rather than from beer production. This choice was motivated by the similarity of the output streams. Results are reported in the Supplementary Figures 3-6 and commented on in the Main Text.

### FIFO Determination

FIFO was calculated according to Jackson (2009):

$$FIFO=\frac{Fish meal level in diet+Fish oil level in diet}{Fish meal yield+Fish oil yield}\times eFCR$$

The fishmeal yield was assumed to be 22.5%, and the fish oil yield was 5% (Jackson, 2009). eFCR (economic FCR = weight of feed fed/animal harvested) was set equal to 1.35, the values for tilapia and other cichlids reported by IAFFD (no date).

The FIFO values for the three diets proposed for replacing BSG in tilapia feeding are 1.32, 1.08, and 0.91 for the Control, D1, and D2 diets, respectively. These values are relatively high compared with the actual FIFO values reported in the literature for tilapia (Glencross et al., 2024). However, they help visualize the significant advantage of using BSG as an FM replacement in terms of fish stock stress.

# Supplementary Figures and Tables

##
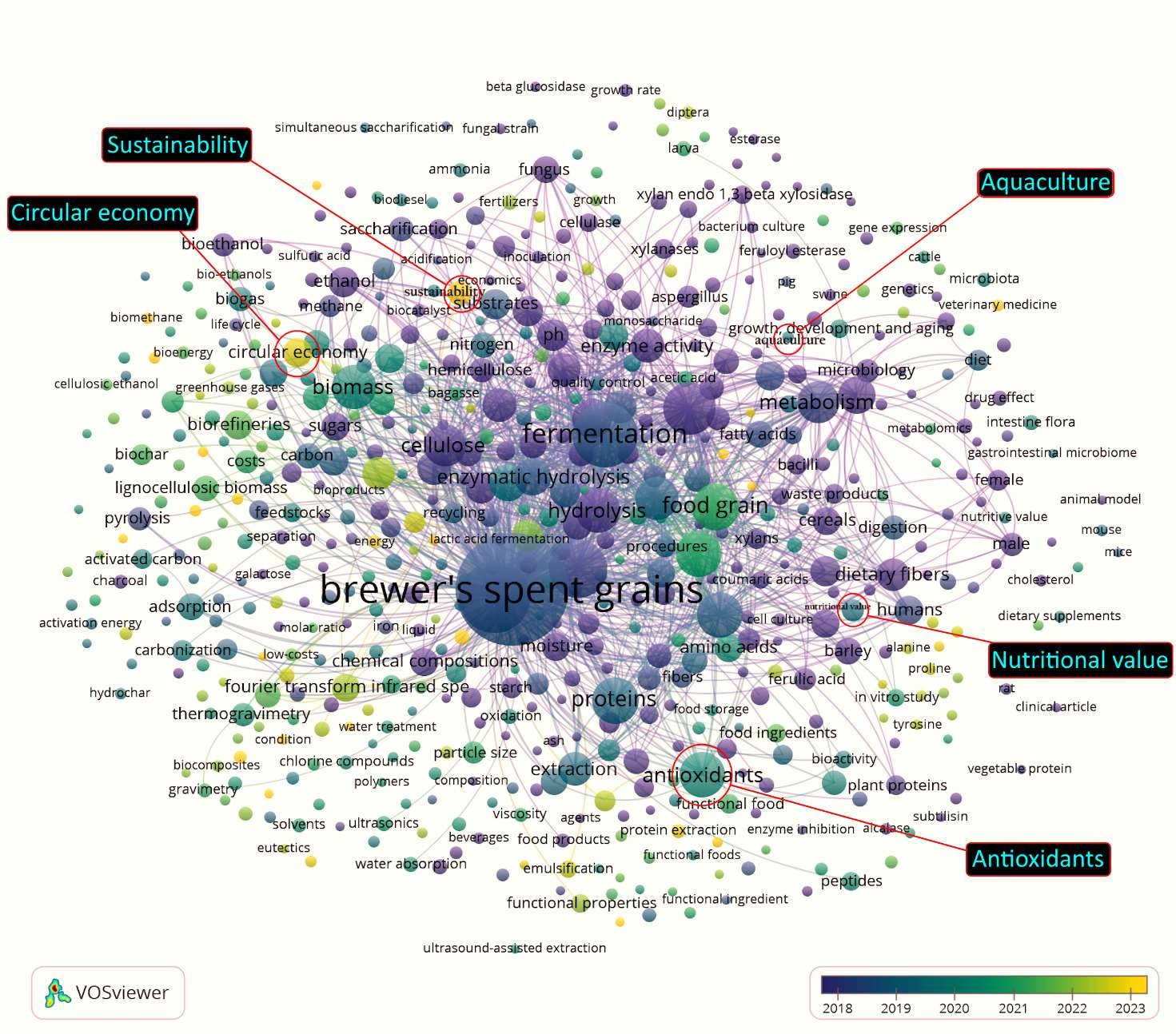
Supplementary Figures

**Supplementary Figure S1.** Bibliometric network analysis map showing the temporal trend of keywords related to the topic "brewer's spent grain" on a set of 1403 documents published from 1953 to 2025, selected using "brewer's spent grain" as a keyword (Vosviewer software, version 1.6.19, applied on the Scopus database). The keywords related to applying BGS in fish feeding are pointed out. Circular economy and sustainability are the most recent subtopics related to BSG (yellowish color).


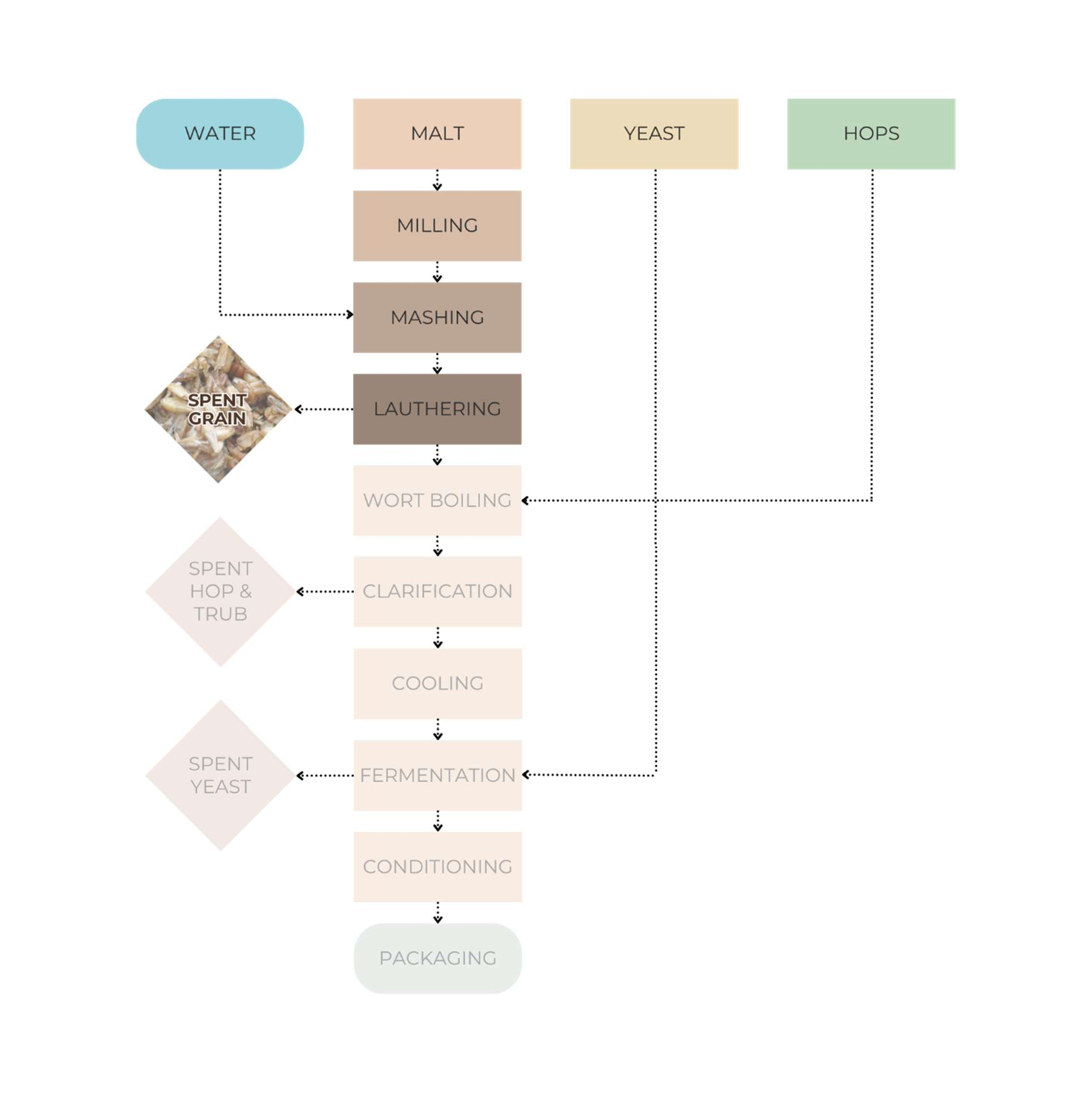


**Supplementary Figure S2.** Flow chart of the production process of beer and the related waste products. The beer production process includes malting, milling, mashing, brewing, cooling, and fermentation. With the mashing process, barley starch is enzymatically converted into fermentable and non-fermentable sugars, while soluble proteins, polypeptides, and amino acids are liberated. A filtration step (lautering) follows, producing a sweet liquid, the wort, containing fermentable sugars that can be converted into ethanol, while the insoluble, undegraded part of the filtrated malted barley grain is the BSG (Xiros and Christakopoulos, 2012).

**Supplementary Figure S3.** LCA results: Relative contribution of Control diet ingredients to environmental impacts.

**Supplementary Figure S4.** LCA results: Relative contribution of D1 diet ingredients to environmental impacts.

**Supplementary Figure S5.** LCA results: Relative contribution of D2 diet ingredients to environmental impacts.

**Supplementary Figure S6.** LCA results: Comparison of the environmental impacts related to the three diets.

## Supplementary Tables

**Supplementary Table S1.** Calculated diet formulation to evaluate the consequences on FIFO and environmental impacts of BSG introduction in fish feeding. Control diet was from Zerai et al. (2008). In D1, 10% BSG allowed the reduction of the diet's fishmeal and wheat flour content. In D2, with 37% BSG, soybean meal was also reduced. The formulation is based on the IAFFD database (IAFFD, no date). CP = crude proteins

| INGREDIENT | Control | D1 | D2 |
| --- | --- | --- | --- |
| Wheat feed flour | 44 | 29.84 | 20.54 |
| Soybean meal 50% CP | 25 | 34.16 | 20 |
| Fish meal 60% CP | 25 | 20 | 17 |
| Fish oil | 2 | 2 | 1.5 |
| Vitamin premix, tilapia | 2 | 2 | 2 |
| Soy Lecithin | 2 | 2 | 2 |
| BSG | 0 | 10 | 36.93 |

**Supplementary Table S2.** Composition (%) of the diets described in Table 1. Based on the IAFFD database (IAFFD, no date).

|  |  | Control | D1 | D2 |
| --- | --- | --- | --- | --- |
|  | Dry matter | 89.47 | 89.69 | 90.56 |
|  | Ash | 6.05 | 6.05 | 5.72 |
|  | Protein | 34.47 | 36.28 | 33.40 |
|  | Carbohydrates | 41.87 | 40.19 | 43.48 |
|  | Lipid | 7.08 | 7.17 | 7.96 |
|  | Fiber | 1.72 | 3.40 | 7.03 |

# Supplementary References

Glencross, B. D., Bachis, E., Robb, D., and Newton, R. (2024). TThe evolution of sustainability metrics for the marine ingredient sector: Moving towards holistic assessments of aquaculture feed. *Rev. Fish. Sci. Aquac.* 0, 1–17. doi: 10.1080/23308249.2024.2337426

Huijbregts, M. A. J., Steinmann, Z. J. N., Elshout, P. M. F., Stam, G., Verones, F., Vieira, M. D. M., et al. (2016). ReCiPe 2016: A harmonized life cycle impact assessment method at midpoint and endpoint level. Bilthoven, The Netherlands. Available at: https://www.rivm.nl/publicaties/recipe-2016-a-harmonized-life-cycle-impact-assessment-method-at-midpoint-and-endpoint (Accessed May 9, 2025).

IAFFD (no date). IAFFD (The International Aquaculture Feed Formulation Database). Available at: https://iaffd.com (Accessed May 6, 2025).

ISO (2006). *Environmental management–Life Cycle Assessment–Principles and framework*. Geneva, (14040:2006): ISO.

Jackson, A. (2009). Fish in - Fish out ratios explained. *Aquaculture Europe* 34, 5–10. Available at: https://www.iffo.com/system/files/downloads/EAS%20FIFO%20September2009%202_0.pdf (Accessed May 6, 2025).

Xiros, C., and Christakopoulos, P. (2012). Biotechnological potential of brewers spent grain and its recent applications. *Waste Biomass Valor.* 3, 213–232. doi: 10.1007/s12649-012-9108-8

Zerai, D. B., Fitzsimmons, K. M., Collier, R. J., and Duff, G. C. (2008). Evaluation of brewer’s waste as partial replacement of fish meal protein in Nile tilapia, *Oreochromis niloticus*, diets. *J. World Aquac. Soc.* 39, 556–564. doi: 10.1111/j.1749-7345.2008.00186.x
